# Epigenetic Biomarkers in Cardiovascular Diseases

**DOI:** 10.3389/fgene.2019.00950

**Published:** 2019-10-09

**Authors:** Carolina Soler-Botija, Carolina Gálvez-Montón, Antoni Bayés-Genís

**Affiliations:** ^1^Heart Failure and Cardiac Regeneration (ICREC) Research Program, Health Science Research Institute Germans Trias i Pujol (IGTP), Badalona, Spain; ^2^CIBERCV, Instituto de Salud Carlos III, Madrid, Spain; ^3^Cardiology Service, HUGTiP, Badalona, Spain; ^4^Department of Medicine, Barcelona Autonomous University (UAB), Badalona, Spain

**Keywords:** epigenetics, biomarker, microRNA, cardiovascular diseases, myocardial infarction, heart failure, atherosclerosis, hypertension

## Abstract

Cardiovascular diseases are the number one cause of death worldwide and greatly impact quality of life and medical costs. Enormous effort has been made in research to obtain new tools for efficient and quick diagnosis and predicting the prognosis of these diseases. Discoveries of epigenetic mechanisms have related several pathologies, including cardiovascular diseases, to epigenetic dysregulation. This has implications on disease progression and is the basis for new preventive strategies. Advances in methodology and big data analysis have identified novel mechanisms and targets involved in numerous diseases, allowing more individualized epigenetic maps for personalized diagnosis and treatment. This paves the way for what is called pharmacoepigenetics, which predicts the drug response and develops a tailored therapy based on differences in the epigenetic basis of each patient. Similarly, epigenetic biomarkers have emerged as a promising instrument for the consistent diagnosis and prognosis of cardiovascular diseases. Their good accessibility and feasible methods of detection make them suitable for use in clinical practice. However, multicenter studies with a large sample population are required to determine with certainty which epigenetic biomarkers are reliable for clinical routine. Therefore, this review focuses on current discoveries regarding epigenetic biomarkers and its controversy aiming to improve the diagnosis, prognosis, and therapy in cardiovascular patients.

## Introduction

Cardiovascular diseases (CVDs) are one of the leading causes of mortality in developed countries. Cardiovascular diseases refer to disorders affecting the structures or function of the heart and blood vessels, including hypertension, atherosclerosis, myocardial infarction (MI), ischemia/reperfusion injury, stroke, and heart failure (HF), among others (Wang et al., 2016a; Thomas et al., 2018). Mechanisms underlying the complex pathophysiology that leads to CVDs are of great interest but still far from clear. Progress in the field of epigenetics have opened a new world for the comprehension and management of human diseases, including the prevalence of CVDs, based on the role of genetics and its environmental interaction in pathological conditions (Jaenisch and Bird, 2003). Significant evidence suggests that the environment and lifestyle can define epigenetic patterns throughout life. These epigenetic patterns are a cellular memory of further environmental exposure. Epigenetic modifications are reversible, different among cell types, and can potentially lead to disease susceptibility by producing long-term changes in gene transcription (Fraga et al., 2005; Beekman et al., 2010).

**Figure 1:**
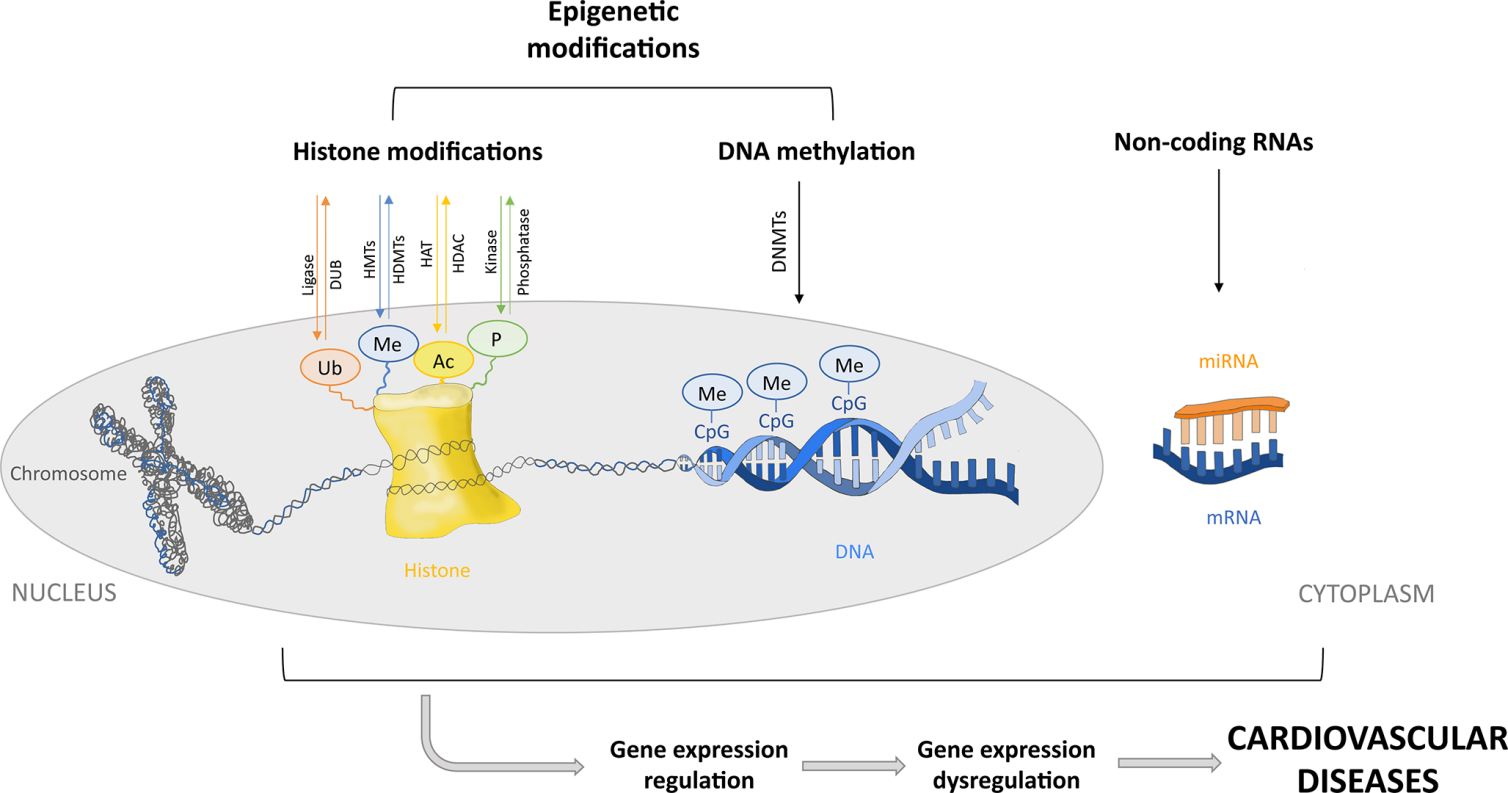
Epigenetic regulatory mechanisms. Posttranslational modifications of histone tails by acetylation, deacetylation, ubiquitination, methylation, and phosphorylation. DNA methylation by DNA methyltransferases (DNMTs). Posttranscriptional regulation of gene expression by microRNAs. Epigenetic modifications involve silencing or downregulation/upregulation of gene expression. Dysregulation of the epigenetic machinery could lead to gene expression dysregulation and cardiovascular diseases. Ubiquitin (Ub), methionine (Me), acetyl group (Ac), phosphate (P), deubiquitinating enzyme (DUB), histone methyltransferase (HMTs), histone demethylase (HDMTs), histone acetyltransferase (HAT), histone deacetylase (HDAC), a cytosine followed by a guanine (CpG), microRNAs (miRNAs), and messenger RNA (mRNA).

Epigenetic modifications include DNA methylation and posttranslational modifications of histone tails. However, in this review, posttranscriptional regulation of gene expression by noncoding RNAs (ncRNAs) is also considered a part of the epigenetic machinery. MicroRNAs (miRNAs) are small ncRNAs that contribute to regulation of the expression of different epigenetic regulators such as DNA methyltransferases (DNMTs) and histone deacetylases (HDACs), among others. Similarly, DNA methylation and histone modifications can regulate the expression of some miRNAs, forming a feedback loop. Thus, miRNAs and epigenetic regulators cooperate to modulate the expression of mutual targets. Therefore, although miRNAs are not strictly considered epigenetic factors, they contribute to the modulation of gene expression through epigenetics. Disruption of this complex regulation may participate in the development of different diseases (Iorio et al., 2010; Hoareau-Aveilla and Meggetto, 2017; Moutinho and Esteller, 2017; Wang et al., 2017a) (**Figure 1**). DNA and histone proteins comprise the chromatin, which can be remodeled into a tightly condensed state (heterochromatin) or an open conformation (euchromatin) that would allow access to transcription factors or DNA binding proteins, allowing the regulation of gene expression (Kouzarides, 2007). Thus, epigenetics involves changes in gene expression due to chromatin adjustments that change the accessibility of DNA without changing its sequence, leading to silencing or downregulation/upregulation of gene expression (Baccarelli et al., 2010). Chromatin modifications, such as DNA methylation, consist of the transfer of a methyl group to carbon 5 of the cytosine residues [5-methylcytosine (5mC)] in CpG dinucleotides sites. CpG dinucleotides are localized throughout the genome but are more abundant in certain regions, such as gene promoters, forming so-called CpG islands. CpG methylation causes transcriptional repression by directly blocking transcription factor access to the DNA or indirectly *via* chromatin-modifying proteins (methyl-binding proteins) that recognize the methylated regions and recruit corepressors. DNA methyltransferases catalyze DNA methylation by recognizing and maintaining hypermethylated DNA during replication (DNMT1) or by *de novo* methylation (DNMT3a and DNMT3b). Moreover, gene bodies of actively transcribed genes normally show slightly higher DNA methylation levels as compared to gene bodies of nontranscribed genes. In contrast, hypomethylation is usually found in enhancer regions and promoters (Costantino et al., 2018). Posttranslational modification of histone tails is another epigenetic modification that regulates gene expression by chromatin remodeling. Histone acetylation, deacetylation, methylation, phosphorylation and ubiquitination change DNA accessibility, regulating gene transcription. The acetylation of histone tails is regulated by histone acetyltransferases (HATs) and HDACs. Histone acetyltransferase enzymes acetylate the lysine residues of the histones, whereas HDACs deacetylate them, promoting gene activation or silencing, respectively. Histone methylation is regulated by histone methyltransferases (HMTs) and histone demethylases (HDMT). Methylation occurs at the lysine or arginine residues and can activate or repress gene transcription depending on the degree of methylation and which residue is methylated (Li et al., 2017c; Sabia et al., 2017). The serine, threonine, and tyrosine residues of histone tails can also be phosphorylated and dephosphorylated by protein kinases and phosphatases, respectively. Histone tail phosphorylation modulates chromatin structure, taking part in transcription, DNA repair, and chromatin compaction in cell division and apoptosis (Rossetto et al., 2012). Lastly, histone tail ubiquitination is sequentially catalyzed by ligases enzymes, which attach ubiquitin to lysine residues. Ubiquitination and deubiquitination are involved in the activation of transcription and are usually associated with histone methylation. Their effect on repressing or activating transcription generally depends on what histone is modified (Cao and Yan, 2012). Finally, miRNAs regulate gene expression *via* degradation of the transcript or repression of translation when binding to the 3′-untranslated region of the target mRNA. Thus, miRNA represses mRNA translation without changing the DNA sequence of the gene. MicroRNA binding to mRNA is imperfect, so each miRNA has multiple targets. This allows the regulation of a great part of the human genome (Bartel, 2009). The miRNAs are 19-25 nucleotides in length, encoded in the genome and transcribed into primary miRNA (pri-miRNA). Pri-miRNAs derive into miRNAs precursors (pre-miRNA) by the nuclear RNase III called Dorsha and are transferred to the cytoplasm and processed by the endonuclease Dicer to generate a double-stranded miRNA duplex. This product is incorporated into an RNA-induced silencer complex (RISC)–loading complex. Then, one strand is removed from the complex, and the other strand forms a mature RISC, serving as a template for target mRNAs (Sato et al., 2011; Nishiguchi et al., 2015).

Due to this important function in gene regulation, epigenetic modifications and miRNA may play a crucial role in the development of pathological conditions, including CVDs. Understanding the epigenetic machinery underlying cardiac disorders and how these epigenetic mechanisms can be introduced into diagnostics (i.e., biomarkers) and therapies is fundamental to improving the quality of life of patients. In medicine, a biomarker is defined as a measurable characteristic that indicates a particular physiological or pathological state or a response to a therapeutic treatment (Strimbu and Tavel, 2010). Ideally, biomarkers should have easy accessibility, predictable detection, and reliability (Sun et al., 2017). It is mandatory to present a specific measurable change that clearly associates with a diagnosis or a predictable outcome. Thus, biomarkers provide information to physicians when evaluating the probability of developing a disease, making a diagnosis, evaluating the severity of a disease and its progression; during therapeutic decision making; or when monitoring a patient’s response and may result in significant cost reduction (Baccarelli et al., 2010). Their classification can be based on their application (predisposition, diagnosis, monitoring, safety, prognostic, or predictive biomarkers). Predisposition biomarkers determine how likely it is for a patient to develop a certain disease and are usually utilized when there is a personal or family history that indicates a disease risk, and the results can help guide medical care. Diagnostic biomarkers are used to detect or confirm the existence of a health disorder and may assist its early detection. Monitoring biomarkers evaluate the status of a disease or determine exposure to an environmental agent or medical product. Safety biomarkers indicate the probability, presence, or extent of toxicity of a certain medical product or environmental agent. Prognostic biomarkers indicate how a disease may progress in patients who already have the particular disease. These biomarkers do not predict the treatment response but can be useful when selecting patients for treatment. Predictive biomarkers identify patients who are most likely to have a favorable or unfavorable response to a specific treatment. Thus, they can predict treatment success or undesired side effects in a particular patient. A particular disease can have different biological mechanisms in different patients. Predictive biomarkers can be associated with the specific mechanism of a health disorder. This facilitates a targeted therapy, which uses drugs specific for a particular biological mechanism associated with a disease, increasing its effectiveness (FDA-NIH Biomarker Working Group, 2016). Specifically, epigenetic biomarkers belonging to most of these classifications are discussed in this review, with a focus on CVDs. Among the epigenetic biomarkers, miRNAs are the most attractive, as they can be detected in small sample volumes, are stable, and can be obtained from plasma, serum, saliva, and urine. Interestingly, they are highly conserved, and this allows a reliable comparison between patients and animal models of disease (Matsumoto et al., 2013). Therefore, although all epigenetic mechanisms are being intensively investigated, miRNAs are evaluated the most for their use as predictive biomarkers. This review presents an overview of current research on epigenetic biomarkers in CVDs and how this knowledge can benefit the diagnosis, prognosis, and therapy for cardiovascular patients.

## Epigenetic Biomarkers in CVDs

Over the last few years, numerous studies have linked cardiovascular risk factors to epigenetic modifications in human patients. Modification of the epigenetic environment alters cardiovascular homeostasis and impacts cardiovascular disorders. The function of epigenetic mechanisms in the regulation of gene expression is well known, although the role of epigenetic marks in CVDs is not clearly understood. Thus, the exploration of epigenetic biomarkers may lead to a deep comprehension of the molecular mechanisms and pathways associated with CVDs. In this section, we focus on major CVDs, such as hypertension, atherosclerosis, MI, and HF, and the epigenetic biomarkers associated with them.

### Hypertension

**Figure 2:**
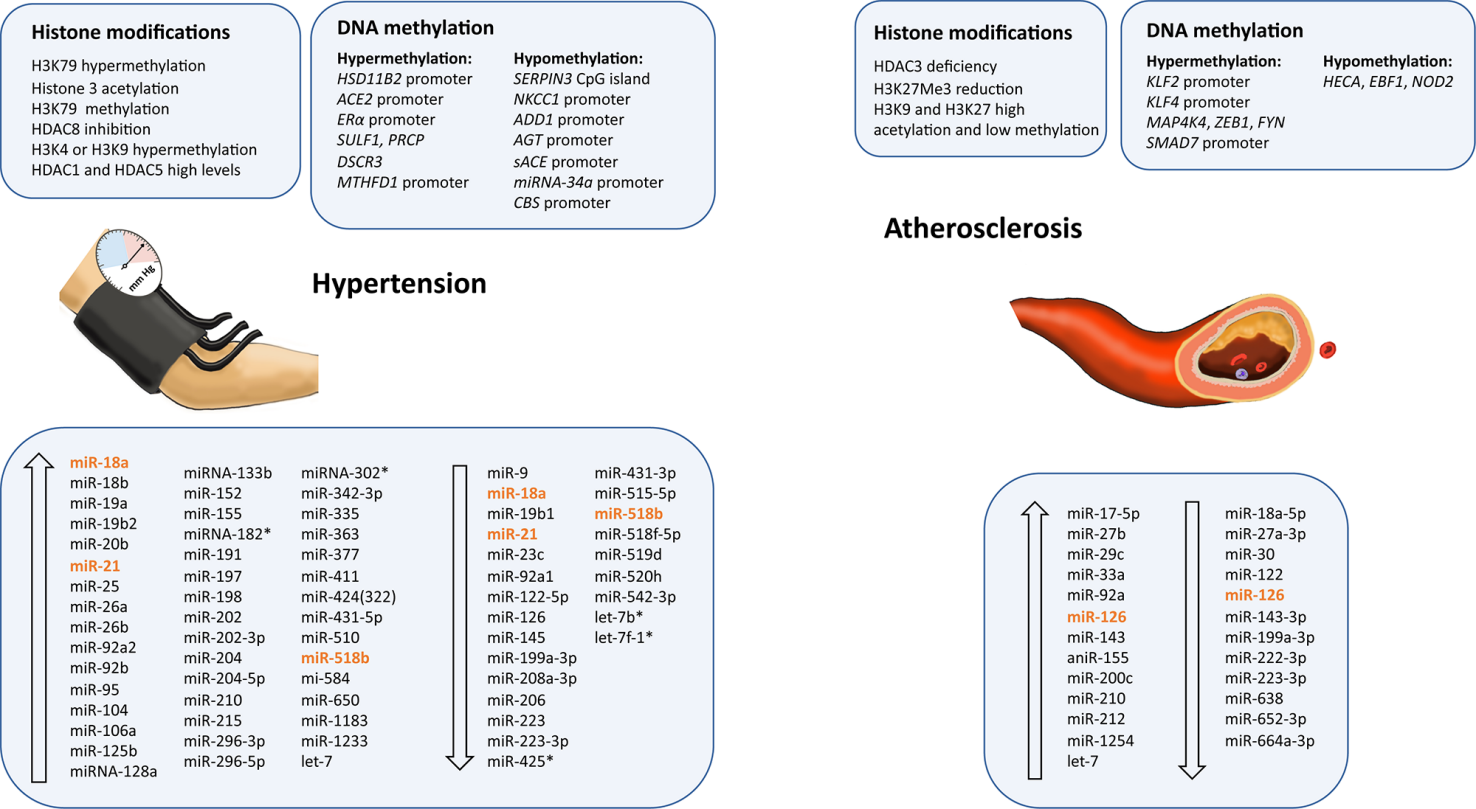
Epigenetic modifications and microRNAs biomarkers dysregulated in atherosclerosis and hypertension. Ascending arrows indicate higher levels or upregulation, and descending arrows denote lower levels or downregulation, both compared to control conditions. Those miRNAs presenting opposite results are shown in orange.

Arterial hypertension is a multifactorial disease with several mechanisms and metabolic systems involved in its pathogenesis. Genetic factors and environmental background may lead to alterations in multiple pathways that can eventually trigger development of the disease (Franceschini and Le, 2014). Intrauterine alterations, such as malnutrition, starvation, obesity, alcohol, drugs, nicotine, or environmental toxins, are some of the environmental factors directly related to hypertension development in the progeny (Bogdarina et al., 2007; Nuyt and Alexander, 2009). In addition, individuals who have aerobic training present with lower blood pressure than nontrained individuals (Fagard, 2006). This has an important impact on CVD risk factor control and is a nonpharmacological way to treat patients. There are also epigenetic factors that can influence the appearance of hypertension in adults, such as hypermethylation of genes, including superoxide dismutase-2 (*SOD2*) or *Granulysin*, or increased levels of histone acetylation at the promoter of the endothelial oxide synthetase gene (*eNOS*) (Wang et al., 2018b). Environmental factors are important to determining an individual’s predisposition to developing major cardiovascular risk factors by means of epigenetic modifications, and identification of the epigenetic mechanisms that participate in hypertension development may help generate new treatments. This is of great interest because hypertension is a key risk factor for CVDs, including MI, HF, stroke, and end-stage renal disease (**Table 1** and **Figure 2**).

**Table 1:** Epigenetic biomarkers in hypertension.

Epigenetic modification	Biomarker	Regulation in hypertension	Sample source	Study type	References
**DNA methylation**	*HSD11B2* promoter	Highly methylated	Rat’s urine and tissues and human cell lines	Experimental: *in vitro* and rat model	(Alikhani-Koopaei et al., 2004)
	*SERPIN3* CpG island	Hypomethylation	Placental tissue	Clinical	(Chelbi et al., 2007)
	*HSD11B2* promoter	Highly methylated	Blood and urine	Clinical	(Friso et al., 2008)
	5mC	Lower levels	Blood	Clinical	(Smolarek et al., 2010)
	*NKCC1* promoter	Hypomethylation	Aorta, heart and kidney	Experimental: spontaneously hypertensive rodent model	(Lee et al., 2010; Cho et al., 2011)
	*sACE* promoter	Hypermethylation	Blood	Clinical and experimental: *in vitro*	(Rivière et al., 2011)
	ERα promoter	Methylation	Uterine arteries	Clinical	(Dasgupta et al., 2012)
	*SULF1*, *PRCP*	*SULF1*: hypermethylation; *PRCP*: hypomethylation	Blood	Clinical	(Wang et al., 2013b)
	*ADD1* promoter	Hypomethylation	Plasma	Clinical	(Zhang et al., 2013a)
	5mC, 5hmC	Higher levels	Tissue	Experimental: Dahl salt-sensitive rats	(Liu et al., 2014)
	*AGT* promoter	Demethylation	H295R cells and visceral adipose tissue	Experimental: *in vitro* and rat model	(Wang et al., 2014a)
	DSCR3	Hypermethylation	Maternal blood and placental tissue	Clinical	(Kim et al., 2015)
	*miRNA-34a* gene promoter	Hypomethylation	Placental tissue	Clinical	(Rezaei et al., 2018)
	*ACE2* promoter	Hypermethylation	Plasma	Clinical	(Fan et al., 2017)
	*CBS* promoter	Hypermethylation	Maternal blood and placental tissue	Clinical	(Kim et al., 2015)
	*MTHFD1* promoter	Hypermethylation	Plasma	Clinical	(Xu et al., 2019)
**Histone modifications**	H3K79	Hypermethylation	NA	Clinical	(Rodriguez-Iturbe, 2006; Duarte et al., 2012)
	Histone 3	Acetylation	Germ cells	Review	(Irmak and Sizlan, 2006)
	H3K79	DNA methylation	Bibliography	Review	(Zhang et al., 2009)
	HDAC8	Inhibition	mDCT cells and tissues	Experimental: rat models of salt-sensitive hypertension	(Mu et al., 2011)
	H3K4 or H3K9	Hypermethylation	Tissue, plasma, and urine	Experimental: LSD1 knockout mice with a high-salt diet	(Pojoga et al., 2011)
	HDAC1, HDAC5	High levels	Lung tissue and adventitial fibroblasts	Clinical and experimental: *in vitro* and hypoxic rat	(Zhao et al., 2012)
**miRNA**	miR-18a, miR-210, miR-152, miR-363, miR-377, miR-411, miR-518b, miR-542-3p	miR-18a, miR-363, miR-377, miR-411, miR-542-3p: underexpression; miR-210, miR-152, miR-518b: overexpression	Placental tissue	Clinical	(Zhu et al., 2009)
	22 miRNAs	15 upregulated and 7 downregulated	Serum	Clinical	(Yang et al., 2011)
	let-7b, miR-302*, miR-104, miR-128a, miR-182*, miR-133b	Overexpression	Placental tissue	Clinical	(Noack et al., 2011)
	miR-92b, miR-197, miR-342-3p, miR-296-5p, miR-26b, miR-25, miR-296-3p, miR-26a, miR-198, miR-202, miR-191, miR-95, miR-204, miR-21, miR-223	miR-92b, miR-197, miR-342-3p, miR-296-5p, miR-26b, miR-25, miR-296-3p, miR-26a, miR-198, miR-202, miR-191, miR-95, miR-204: overexpression; miR-21, miR-223: underexpression	Placental tissue	Clinical	(Choi et al., 2013)
	miR-9, miR-126	Lower levels	Peripheral blood mononuclear cells	Clinical	(Kontaraki et al., 2014)
	miR1233	Higher levels	Serum	Clinical	(Ura et al., 2014)
	miR-18a, miR-19b1, miR-92a1, miR-210	miR-210: upregulation; miR-18a, miR-19b1, and miR-92a1: downregulation	Plasma and placental tissue	Clinical	(Xu et al., 2014)
	miR-505	Upregulation	Plasma	Clinical	(Yang et al., 2014)
	miR-106a, miR-18b, miR-20b, miR-19b-2, miR-92a-2, miR-363	Dysregulation	Placental tissue	Clinical	(Zhang et al., 2015a)
	miR-515-5p, miR-518b, miR-518f-5p, miR-519d, miR-520h	Downregulation	Placental tissue	Clinical	(Hromadnikova et al., 2015)
	miR-335, miR-584	Upregulation	Placental tissue and HTR8/Svneo cells	Clinical and experimental: *in vitro*	(Jiang F. et al., 2015)
	miR-125b	Overexpression	Plasma and placental tissue	Clinical	(Yang et al., 2016b)
	miR-215, miR-155, miR-650, miR-210, miR-21, miR-18a, miR-19b1	MiR-215, miR-155, miR-650, miR-210, miR-21: upregulation; miR-18a, miR-19b1: downregulation	Plasma	Clinical	(Jairajpuri et al., 2017)
	miR-204-5p	Higher levels	Serum	Clinical	(Mei et al., 2017)
	let-7b*, let-7f-1*, miR-1183, miR-23c, miR-425*	miR-1183: upregulation; let-7b*, miR-23c, miR-425*, let-7f-1*: downregulation	Plasma and placental tissue	Clinical	(Gunel et al., 2017)
	miR-145	Downregulation	Placental tissue	Clinical	(Han et al., 2017)
	miR-202-3p	Upregulation	Placental tissue	Clinical	(Singh et al., 2017)
	let-7	Higher	Plasma	Clinical	(Huang et al., 2017b)
	miRNA	Dysregulation	Bibliography: Maternal serum and placental tissue	Bibliography review	(Laganà et al., 2018)
	miR-19a	Upregulation	Plasma and lung tissue	Clinical	(Chen and Li, 2017)
	miR-21	Upregulation	Peripheral blood mononuclear cells	Clinical	(Parthenakis et al., 2017)
	miR-21	Upregulation	Bibliography review	Bibliography review	(Sekar et al., 2017)
	miR-510	Upregulation	Serum	Clinical	(Krishnan et al., 2017)
	miR-206	Lower levels	Serum	Clinical	(Jin et al., 2017)
	miR-424(322)	Upregulation	Plasma	Clinical	(Baptista et al., 2018)
	miR-199a-3p, miR-208a-3p, miR-122-5p, miR-223-3p	Downregulation	Serum	Clinical	(Zhang et al., 2018c)
	miR-431-5p	Upregulation	Tissue	Experimental: mice made hypertensive and *in vitro*	(Huo et al., 2019)
	miR-143, NR_034083, NR_104181,	miR-143: upregulation; NR_034083: downregulation and NR_104181 and	Peripheral blood leucocytes	Clinical	(Chen et al., 2018b)

NA, not available.

Essential hypertension is a multifactorial disease with no identifiable cause that is affected by environmental and epigenetic factors. Environmental stressors cause acetylation of histone 3 in the neurons of the area postrema, leading to an increase in pressure that results in hypertension (Irmak and Sizlan, 2006). Low activity of the 11 beta-hydroxysteroid dehydrogenase 2 (HSD11B2) induces hypertension. In a study performed in patients with essential hypertension or glucocorticoid-induced hypertension, the *HSD11B2* promoter was highly methylated. These changes may reflect a global status, with methylation of gene promoter being a potentially useful molecular biomarker to characterize hypertensive patients (Alikhani-Koopaei et al., 2004; Friso et al., 2008). Moreover, a polymorphism in the disruptor of telomeric silencing-1 gene (*DOT1L*), which encodes a methyltransferase that enhances methylation of histone 3 (H3K79) in the renal epithelial sodium channel gene (*ENaC*) promoter, is associated with blood pressure regulation (Duarte et al., 2012). It has also been reported that a *DOT1A* and *ALL1* (fused gene from chromosome 9 [Af9]) interaction is associated with H3K79 hypermethylation of the *ENaC* promoter, suppressing its transcriptional activity. This interaction is disrupted by aldosterone and causes hypomethylation of H3K79 at specific regions, disinhibiting the *ENaC* promoter and leading to hypertension. Thus, the Dot1a-Af9 pathway may also be involved in the control of genes implicated in hypertension (Zhang et al., 2009). Hypomethylation of the α-adducin gene (*ADD1*) promoter has been found to be connected to the risk of essential hypertension. However, differences between females and males have been found (Zhang et al., 2013a). Moreover, histone 3 (H3K4 or H3K9) demethylation is induced by lysine-specific demethylase-1 (LSD1), which modifies gene transcription. Hypermethylation of histone 3 has been associated with hypertension, increased vascular contraction, and decreased relaxation *via* the nitric oxide-cGMP (NO-cGMP) pathway in heterozygous *LSD1* knockout mice fed a high-salt diet (Pojoga et al., 2011). Histone deacetylation is also important in the development of pulmonary arterial hypertension. HDAC1 and HDAC5 protein levels have been demonstrated to be elevated in the lungs of patients and hypoxic rats. Inhibition of these proteins by valproic acid and suberoylanilide hydroxamic acid diminished the development of hypoxia-induced pulmonary hypertension in rats. Thus, HDAC1 and HDAC5 levels could be useful predictive biomarkers for the treatment of pulmonary hypertension in patients (Zhao et al., 2012).

In a study evaluating alterations in the global DNA methylation status of patients with essential hypertension, the level of the epigenetic marker 5mC was lower in hypertensive patients than in healthy people (Smolarek et al., 2010). In an *in vivo* model of hypertension using Dahl salt-sensitive rats, the levels of 5mC and 5-hydroxymethylcytosine (5hmC) were evaluated in the outer renal medulla. In response to salt administration, the 5mC levels were significantly higher for genes with low transcription and 5hmC levels higher in genes with higher expression. This study revealed important features of 5mC and 5hmC for understanding the role of epigenetic modifications in the regulation of hypertension (Liu et al., 2014).

Rivière et al. (2011) analyzed the regulation of somatic angiotensin-converting enzyme gene (*sACE*) expression by promoter methylation. *sACE* regulates blood pressure by catalyzing the conversion of angiotensin I into angiotensin II, a potent vasopressor. Hypermethylation of *sACE* promoter in cultures of human endothelial cells and rats was associated with transcriptional repression, suggesting an epigenetic mechanism in hypertension regulation (Rivière et al., 2011). More recently, Fan et al. (2017) demonstrated opposite results in patients with essential hypertension. The authors indicated that hypermethylation of the *ACE2* promoter may increase essential hypertension risk, with variabilities in CpG islands methylation in males and females (Fan et al., 2017).

Moreover, a genome-wide methylation study on essential hypertension revealed that changes in the DNA methylation of leukocytes are involved in the pathogenesis of hypertension. They found increased methylation in the gene encoding sulfatase 1 (*SULF1*), which is involved in apoptosis, and decreased methylation in the gene encoding prolylcarboxypeptidase (*PRCP*), a regulator of angiotensin II and III cleavage (Wang et al., 2013b). Another genome-wide study of blood pressure characteristics found new genetic variants that influence blood pressure and are strongly associated with local CpG island methylation. This study demonstrated the role of DNA methylation in the regulation of blood pressure (Kato et al., 2015).

The pathogenesis of hypertension is affected by alterations in ion flux mechanisms. Hypomethylation of the Na/K/2Cl cotransporter 1 gene (*NKCC1*) promoter results in overexpression in a rodent model with spontaneous hypertension (Lee et al., 2010). DNA methyltransferase activity maintained hypomethylation in the *NKCC1* promoter, playing an important role in *NKCC1* upregulation during the course of the disease. This encourages evaluation of the *NKCC1* methylation status in hypertensive patients (Cho et al., 2011). Furthermore, WNK4 is a serine-threonine kinase that negatively regulates the Na(+)-Cl(−)-cotransporter (NCC) and ENaC. This would affect the distal nephron, increasing the reabsorption of sodium. Stimulation of β(2)-adrenergic receptor (β(2)AR) in salt intake conditions would reduce *WNK4* transcription, resulting in inhibition of HDAC8 activity and increased histone acetylation. In the rat models of salt-sensitive hypertension, salt diet repressed renal WNK4 expression, activating the NCC and inducing salt-dependent hypertension. Thus, *WNK4* transcription is epigenetically modulated in the course of salt-sensitive hypertension, with the β(2)AR-WNK4 pathway as a potential therapeutic target for this disease (Mu et al., 2011).

Goyal et al. (2010) demonstrated that a low protein diet in pregnant mice leads to alterations in DNA methylation, miRNA, and gene expression in the brain renin–angiotensin system, a key regulator of hypertension in adults (Goyal et al., 2010). Along the same lines, in a study carried out *in vitro* and in a rat model, DNA demethylation of the angiotensinogen gene (*AGT*) promoter activated its expression. AGT is an important substrate of the renin–angiotensin–aldosterone system and an important target in hypertension research. Elevated concentrations of circulating aldosterone and high consumption of salt stimulate the AGT gene expression in adipose-induced hypertension (Wang et al., 2014a). In addition, cystathionine β-synthase (CBS), an important enzyme in the metabolism of plasma homocysteine, is associated with hypertension and stroke. Hypermethylation of the *CBS* promoter has been demonstrated to increase the risk of both diseases, especially in male patients (Wang et al., 2019a). Similarly, hypermethylation of the methylenetetrahydrofolate dehydrogenase 1 gene (*MTHFD1*) promoter, which is also associated with homocysteine metabolism, was observed in hypertensive patients, and proposed as a potential diagnostic biomarker in patients with essential hypertension (Xu et al., 2019).

In addition to the previous classic epigenetic modifications, miRNAs often regulate hypertension and are attractive biomarkers for the disease. The miR-9 and miR-126 expression levels are significantly lower in hypertensive patients than healthy individuals and are related to hypertension prognosis and organ damage. Thus, miR-9 and miR-126 may be possible biomarkers in essential hypertension (Kontaraki et al., 2014). Moreover, ncRNAs, such as miR-143, miR-145, and NR_104181, are significantly higher in essential hypertensive patients than controls, whereas NR_027032 and NR_034083 are significantly reduced. After evaluating cardiovascular risk factors, they concluded that lower expression levels of NR_034083 and higher expression levels of NR_104181 and miR-143 were risk factors for essential hypertension (Chen et al., 2018b). Another study evaluated the correlation between miRNA let-7 expression and subclinical atherosclerosis in untreated patients with newly diagnosed essential hypertension and found increased levels in hypertensive patients, suggesting that plasma let-7 could be an indicator for monitoring end-organ damage and a biomarker for atherosclerosis in these patients (Huang et al., 2017b). Similarly, upregulation of miR-505, miR-19a, miR-21, miR-510, or miR-424(322) in blood from hypertensive patients suggests a possible use for miR-510 as a diagnostic biomarker and therapeutic target (Yang et al., 2014; Chen and Li, 2017; Krishnan et al., 2017; Parthenakis et al., 2017; Sekar et al., 2017; Baptista et al., 2018). Lower levels of the combination of miR-199a-3p, miR-208a-3p, miR-122-5p, and miR-223-3p have also been shown to be suitable for diagnosis of hypertension (Zhang et al., 2018c). Decreased miR-206 levels might also be especially useful in the detection of pulmonary hypertension in patients with left heart disease (Jin et al., 2017). Furthermore, a study in hypertensive mice produced by infusion of angiotensin II concluded that miR-431-5p knockdown delays the increase in blood pressure induced by angiotensin II and reduces vascular injury. This demonstrates its potential as a target for the treatment of hypertension and vascular injury (Huo et al., 2019).

Preeclampsia is an important pregnancy-induced syndrome characterized by hypertension and proteinuria. Chronic hypoxia is a common pregnancy stress that increases the risk of preeclampsia and is associated with changes in methylation of the estrogen receptor α gene (*ERα*) promoter. ERα is involved in adjustments to the uterine blood flow, and promoter methylation results in gene repression in uterine arteries, increasing blood pressure (Dasgupta et al., 2012). Preeclampsia also modifies the expression profile of several serine protease inhibitors (SERPINs) in the placenta. Specifically, *SERPIN3* CpG islands have a significantly low level of methylation in preeclampsia, providing a new potential marker for early diagnosis (Chelbi et al., 2007). Another study demonstrated a positive association between placenta global DNA methylation and hypertension in preeclampsia (Kulkarni et al., 2011). Next-generation sequencing technology and microarray assay analyses of the miRNA expression pattern in preeclamptic placentas versus healthy placentas have revealed that miRNAs expression is dysregulated in preeclampsia (Zhu et al., 2009; Noack et al., 2011; Yang et al., 2011; Choi et al., 2013; Xu et al., 2014; Hromadnikova et al., 2015; Zhang et al., 2015a; Gunel et al., 2017; Han et al., 2017). These results were in agreement with those found in the miRNA database from cell and tissue analyses. Thus, circulating miRNAs in the serum of pregnant women could be used as biomarkers for the diagnosis and prognosis of preeclampsia. To further demonstrate that miRNAs could be good predictors of preeclampsia, as well as its severity, circulating miRNA signatures were evaluated in women divided into groups based on preeclampsia severity. MiR-21, miR-29a, miR-125b, miR-155, miR-202-3p, miR-204-5p, miR-210, miR-215, miR-335, miR-518b, miR-584, miR-650, and miR-1233 were upregulated, whereas miR-15b, miR-18a, miR-19b1, and miR-144 were downregulated in women with severe preeclampsia compared to mild preeclampsia (Ura et al., 2014; Jiang et al., 2015; Yang et al., 2016b; Jairajpuri et al., 2017; Mei et al., 2017; Singh et al., 2017). In addition, a recent data recompilation supported a direct association between high or low expression of miRNAs in pregnancy serum and placenta in preeclamptic pregnancies (Laganà et al., 2018). Interestingly, an association has also been demonstrated between hypomethylation of the miR-34a promoter and preeclampsia severity (Rezaei et al., 2018). Another study analyzed the concentrations of Down syndrome critical region 3 (*DSCR3*), Ras association domain family 1 isoform A (*RASSF1A*), and sex-determining region Y (*SRY*) cell-free fetal DNA in maternal plasma from preeclamptic pregnancies and found that all of the markers significantly correlated with gestational age. The authors demonstrated that *DSCR3* is a novel epigenetic biomarker and an alternative to *RASSF1A* for the prediction of early-onset preeclampsia (Kim et al., 2015). However, no association was found between the methylation status of the cortisol-controlling gene (*HSD11B2*), tumor suppressor gene (*RUNX3*), or long interspersed nucleotide element-1 gene (*LINE-1*) and hypertensive disorders of pregnancy when placental DNA methylation was analyzed (Majchrzak-Celińska et al., 2017).

### Atherosclerosis

Atherosclerosis is a chronic inflammatory disease characterized by the accumulation of cholesterol in the walls of large- and medium-sized arteries, the accumulation of extracellular matrix and lipids, and smooth muscle cell proliferation. This process leads to the infiltration of immune cells (mostly macrophages) and endothelial dysfunction, forming a plaque, and eventually developing into acute cardiovascular events, such as MI, peripheral vascular disease, aneurysms, and stroke (Wissler, 1991). Proatherogenic stimuli, such as low-density lipoprotein (LDL) cholesterol and oxidized LDL, have been suggested to stimulate a long-term epigenetic reprogramming of innate immune system cells. This induces a constant activation, even after the removal of atherosclerotic stimuli (Bekkering et al., 2016). Emerging evidence supports epigenetic modifications being involved in the initiation and progression of atherosclerosis, playing an important role in plaque development and vulnerability, and highlighting the importance of epigenetic biomarkers as predictors of CVDs (**Table 2** and **Figure 2**) (Xu et al., 2018).

**Table 2:** Epigenetic biomarkers in atherosclerosis.

Epigenetic modification	Biomarker	Regulation in atherosclerosis	Sample source	Study type	References
**DNA methylation**	*KLF2* promoter	Methylation	HUVEC cells	Experimental: *in vitro*	(Kumar et al., 2013)
	*KLF4* promoter	Methylation	HAEC cells	Experimental: *in vitro*	(Jiang et al., 2014)
	*HECA*, *EBF1*, *NOD2*, *MAP4K4*, *ZEB1*, *FYN*	*HECA*, *EBF1*, *NOD2*: Hypomethylated; *MAP4K4*, *ZEB1*, *FYN*: Hypermethylated	Human aortic intima and HEK293 cells	Clinical and experimental: *in vitro*	(Yamada et al., 2014)
	*TIMP1*, *ABCA1*, *ACAT1* promoters	Altered methylation status	Peripheral blood	Clinical	(Ma et al., 2016)
	*SMAD7* promoter	Hypermethylation	Peripheral blood and atherosclerotic plaques	Clinical	(Wei et al., 2018)
	5mC, 5-hmC	Higher levels	Peripheral blood	Clinical	(Jiang et al., 2019)
**Histone modifications**	HDAC3	Deficiency	Aorta and HUVEC cells	Experimental: apoE−/− mice and *in vitro*	(Zampetaki et al., 2010)
	H3K27Me3	Reduction in H3K27Me3 modification	Perirenal aortic tissue patches	Clinical	(Wierda et al., 2015)
	H3K9, H3K27	Higher histone acetylation and lower histone methylation	Carotid tissue	Clinical	(Greißel et al., 2016)
**miRNA**	miR-130a, miR-27b, miR-210	Higher levels	Serum and intima tissue	Clinical	(Li et al., 2011)
	miR-17-5p	Higher levels	Plasma	Clinical	(Chen et al., 2015a)
	miR-143-3p, miR-222-3p	Lower levels	Microparticles	Clinical	(de Gonzalo-Calvo et al., 2016)
	miR-30	Lower levels	Plasma	Clinical	(Huang et al., 2016b)
	miR-92a	Higher levels	Plasma	Clinical	(Huang et al., 2017a)
	miR-18a-5p, miR-27a-3p, miR-199a-3p, miR-223-3p, miR-652-3p	Lower levels	Plasma	Clinical	(Vegter et al., 2017)
	miR-33a	Higher levels	Plasma	Clinical	(Kim et al., 2017)
	miR-126	Lower levels	Plasma	Experimental: apoE−/− mice	(Hao and Fan, 2017)
	miR-212	Overexpression	Serum	Clinical	(Jeong et al., 2017)
	miRNA let-7	Higher levels	Plasma	Clinical	(Huang et al., 2017b)
	miR-1254	Higher levels	Plasma	Clinical	(de Gonzalo-Calvo et al., 2018)
	miR-200c	Overexpression	Carotid plaques and plasma	Clinical	(Magenta et al., 2018)
	miR-29c	Higher levels	Plasma	Clinical	(Huang et al., 2018)
	miR-221, miR-222	Lower expression levels	Serum	Clinical	(Yilmaz et al., 2018)
	miR-638	Lower levels	Serum	Clinical	(Luque et al., 2018)
	miR-122	Higher levels	Serum	Clinical	(Wang and Yu, 2018)
	miR-221, miR-222	Higher levels in tissue samples and lower levels in whole blood	Coronary artery atherosclerotic plaques, and internal mammary arteries and whole blood	Clinical	(Bildirici et al., 2018)
	miR-664a-3p	Downregulation	Serum	Clinical	(Li et al., 2018b)
	miR-155	Higher levels	Serum	Clinical	(Qiu and Ma, 2018)
	miR-19A, miR-19B, miR-126, miR-155	Differential levels	GEO dataset	High throughput	(Mao et al., 2018)
	miR-126, miR-143	Higher levels	Plasma	Clinical	(Gao et al., 2019)

Regarding histone modifications, HDAC3 is reported to have a protective effect in apolipoprotein E deficient (apoE−/−) mice. HDAC3 maintains the endothelial integrity, and its deficiency results in atherosclerosis (Zampetaki et al., 2010). Similarly, increased histone acetylation has been proposed to play some role in the progression of atherogenesis by modulating the expressions of proatherogenic genes (Choi et al., 2005). Histone deacetylases are upregulated in aortic smooth muscle cells when they were stimulated with mitogens. In contrast, inhibition of HDACs reduces aortic smooth muscle cell proliferation by changing cell cycle genes expression. This suggests a protective effect against atherosclerosis (Findeisen et al., 2011). Investigations of the association between changes in lysine 27 trimethylation of histone 3 (H3K27Me3), and atherosclerotic plaque development revealed a reduction in global levels of H3K27Me3 modification in vessels with advanced atherosclerotic plaques. This does not correlate with a reduction in the corresponding HMT, enhancer of zeste homolog 2 (EZH2). There was a relationship between the repression of H3K27Me3 mark in the vessels with advanced atherosclerotic plaques and the dynamic differentiation and proliferation of smooth muscle cells associated with atherosclerotic disease (Wierda et al., 2015). Histone acetylation, methylation, and the expression of their corresponding transferases in the atherosclerotic plaques of patients with carotid artery stenosis have been analyzed. Greißel et al. (2016) analyzed the expression of HATs GCN5L, P300, MYST1, and MYST2 and HMTs MLL2/4, SET7/9, hSET1A, SUV39H1, SUV39H2, ESET/SETDB1, EHMT1, EZH2, and G9a and described an enhancement in histone acetylation on H3K9 and H3K27 in the smooth muscle cells from severe atherosclerotic lesions that correlated with plaque severity. In addition, H3K9 and H3K27 methylation were significantly lower in atherosclerotic plaques and significantly associated with disease severity (Greißel et al., 2016).

DNA methylation is also involved in atherosclerosis. To identify CpG methylation profiles in the progression of atherosclerosis in the human aorta, Valencia-Morales et al. (2015) performed DNA methylation microarray analyses. They detected a correlation between histological pathology and the differential methylation of numerous autosomal genes in vascular tissue, providing potential biomarkers of damage severity and treatment targets (Valencia-Morales et al., 2015). Genes such as *Drosophila* headcase (*HECA*), early B-cell factor 1 (*EBF1*), and nucleotide-binding oligomerization domain containing 2 (*NOD2*) are significantly hypomethylated, whereas mitogen-activated protein kinase kinase kinase kinase 4 (*MAP4K4*), zinc finger E-box binding homeobox 1 (*ZEB1*), and proto-oncogene tyrosine-protein kinase (*FYN*) are hypermethylated in atheromatous plaque lesions compared to the plaque-free intima (Yamada et al., 2014). Another study described differentially methylated regions in genes associated with atherosclerosis in swine aorta endothelial cells (Jiang et al., 2015). Low-density lipoprotein cholesterol risk factor upregulates DNMT1, which methylates and represses the Krüppel-like factor 2 gene (*KLF2*) promoter. KLF2 is a transcription factor essential for endothelium homeostasis, and its repression results in endothelial dysfunction (Kumar et al., 2013). Similarly, DNMT3a upregulation in human aortic endothelial cells exposed to disturbed flow induces the methylation and repression of the Krüppel-like factor 4 gene (*KLF4*) promoter, increasing regional atherosusceptibility (Jiang et al., 2014). In an attempt to determine biomarkers of atherosclerosis in the primary stages, the DNA methylation status was determined in a selection of gene promoters associated with the disease. They analyzed the promoter methylation of ATP binding cassette subfamily A member 1 (*ABCA1*), TIMP metallopeptidase inhibitor 1 (*TIMP1*), and acetyl-CoA acetyltransferase 1 (*ACAT1*) and observed significant alterations in the peripheral blood of atherosclerosis patients (Ma et al., 2016). A recent study found that *SMAD7* expression is decreased and its promoter highly methylated in atherosclerotic plaques compared to normal artery walls. There was also increased DNA methylation of the *SMAD7* promoter in the peripheral blood of atherosclerosis patients. Thus, the *SMAD7* promoter is hypermethylated in atherosclerosis patients and their atherosclerotic plaques, with a positive association with homocysteine levels (Wei et al., 2018). Moreover, increased 5mC and 5-hmC levels, which indicate DNA methylation and hydroxymethylation, respectively, have been demonstrated in peripheral blood mononuclear cells from elderly patients with coronary heart disease. These results positively correlate with the severity of coronary atherosclerosis (Jiang et al., 2019).

MicroRNAs have also been identified as attractive epigenetic biomarkers for atherosclerosis. Li et al. (2011) examined miRNA levels in serum samples and the intima of atherosclerosis obliterans patients and compared them to controls. They observed increased levels of miR-27b, miR-130a, and miR-210 in serum and sclerotic tissue from patients, proposing these miRNAs as epigenetic biomarkers for early stages of the disease (Li et al., 2011). Later, a study with a reduced number of patients suggested that elevated levels of circulating miR-17-5p may be a useful biomarker in the diagnosis of coronary atherosclerosis (Chen et al., 2015a).

Microparticles secreted by human coronary artery smooth muscle cells are a different source of cardiovascular biomarkers. These extracellular vesicles can contain miRNAs, such as miR-21-5p, miR-143-3p, miR-145-5p, miR-221-3p, and miR-222-3p. Lower levels of miR-143-3p and miR-222-3p have been found in microparticles derived from atherosclerotic plaque areas compared to nonatherosclerotic areas (de Gonzalo-Calvo et al., 2016).

Huang et al. (2016b) evaluated the expression of miR-30 in patients with essential hypertension compared to control individuals. They observed a reduction in miR-30 levels in the hypertensive patients and in the increased carotid intima-media thickness group. Thus, the authors suggested that circulating miR-30 may be a useful noninvasive atherosclerosis biomarker for patients with essential hypertension (Huang et al., 2016b). Later, the authors also identified higher levels of miR-92a as a possible biomarker of atherosclerosis in the same type of patients (Huang et al., 2017a).With the aim of investigating correlations between circulating miRNAs specific for HF and atherosclerosis in HF patients, Vegter et al. (2017) assessed miRNAs levels and related them to biomarkers associated with atherosclerotic disease and rehospitalizations of cardiovascular patients. They demonstrated a consistent trend between a high number of atherosclerosis manifestations and lower levels of miR-18a-5p, miR-27a-3p, miR-199a-3p, miR-223-3p, and miR-652-3p. Thus, lower levels of circulating miRNAs in HF patients with atherosclerotic disease and an elevated probability of cardiovascular-related rehospitalization were described (Vegter et al., 2017). High levels of miR-33a have also been demonstrated to be a potential cause of cholesterol accumulation and to exacerbate vessel walls inflammation in atherosclerotic disease. Thus, plasma miR-33a has been proposed as a suitable biomarker in atherosclerosis (Kim et al., 2017).

In an attempt to identify more atherosclerosis biomarkers, Hao and Fan (2017) performed microarray analysis using the plasma from apoE−/− mice and discovered that a reduction in miR-126 levels is a good indicator of atherosclerotic disease. They also determined that miR-126 is involved in the mitogen-associated protein kinase (MAPK) signaling pathway, reducing cytokine release and progressing atherosclerotic pathogenesis (Hao and Fan, 2017). In contrast, Gao et al. (2019) determined that higher expression levels of miR-126 and miR-143 correlate with the presence and severity of cerebral atherosclerosis (Gao et al., 2019). In another study, the authors evaluated the synergy of circulating miRNAs with cardiovascular risk factors to estimate the presence of atherosclerosis in ischemic stroke patients. They identified miR-212 as a novel marker that enhances the estimation of atherosclerosis presence in combination with hemoglobin A_1c_, high-density lipoprotein cholesterol, and lipoprotein(a) (Jeong et al., 2017). Another candidate biomarker for atherosclerosis is miR-200c. The authors analyzed plaque instability in the carotid arteries of patients undergoing carotid endarterectomy by examining the expression of miR-200c. Higher expression of miR-200c positively correlated with instability biomarkers, such as monocyte chemoattractant protein-1, cyclooxygenase-2, interleukin 6 (IL-6), metalloproteinases, and miR-33a/b, and negatively correlated with stability biomarkers, such as ZEB1, endothelial nitric oxide synthase, forkhead boxO1, and Sirtuin1. Thus, miR-200c could be a biomarker of atherosclerotic plaque progression and clinically useful for identifying patients at high embolic risk (Magenta et al., 2018). Along the same lines, lower serum levels of miR-638 may be a suitable biomarker of plaque vulnerability and ischemic stroke in individuals with high cardiovascular risk (Luque et al., 2018). With the intention to explore the role of miRNAs associated with carotid atherosclerosis, Mao et al. (2018) analyzed the genes differentially expressed between primary and advanced atherosclerotic plaques using two public datasets from the Gene Expression Omnibus (GEO) databases. The authors found a total of 23 miRNAs and focused on miR-19A, miR-19B, miR-126, and miR-155, which may be considered biomarkers of carotid atherosclerosis (Mao et al., 2018). In addition, Li et al. (2018b) identified downregulation of specific circulating miR-664a-3p as a biomarker of atherosclerosis in patients with obstructive sleep apnea and enlarged maximum carotid intima-media thickness (Li et al., 2018b).

Circulating miR-221 and miR-222 could also be suitable biomarkers for the diagnosis of atherosclerosis, as lower levels of these miRNAs correlate with the disease (Bildirici et al., 2018; Yilmaz et al., 2018). However, higher levels have been found in samples from coronary atherosclerotic plaques and internal mammary arteries (Bildirici et al., 2018). On the other hand, higher circulating levels of miR-29c, miR-122, and miR-155 in coronary atherosclerosis patients might allow noninvasive detection of the disease and its severity (Huang et al., 2018; Qiu and Ma, 2018; Wang and Yu, 2018). In another interesting study that assessed whether atherosclerosis of different arterial territories, not including the coronary artery, is associated with specific circulating miRNAs, the investigators were able to identify specific miRNA profiles for each territory with atherosclerotic disease. These findings may provide a pathophysiological understanding and be useful for selecting potential biomarkers for clinical practice (Pereira-da-Silva et al., 2018).

### Myocardial Infarction

**Figure 3:**
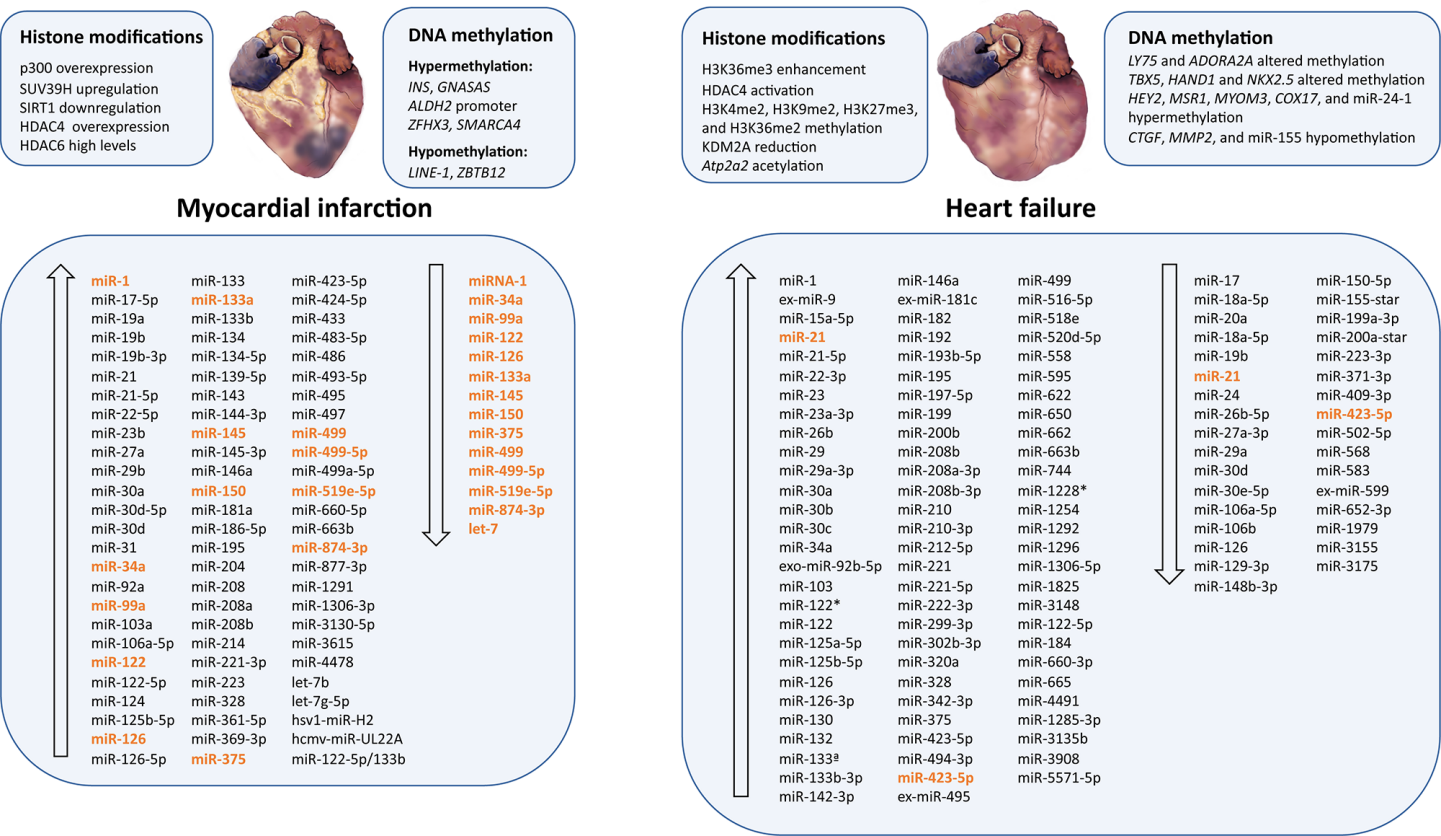
Epigenetic modifications and microRNAs biomarkers dysregulated in myocardial infarction and heart failure. Ascending arrows indicate higher levels or upregulation, and descending arrows denote lower levels or downregulation, both compared to control conditions. Those miRNAs presenting opposite results are shown in orange.

Acute MI (AMI) is a threatening disease worldwide. Early and accurate differential diagnosis is critical for immediate medical intervention and improved prognosis (Reed et al., 2017). In particular, it is important to notice that patients with ST-segment elevation MI (STEMI) have different requirements than patients with non-STEMI (NSTEMI). For the first group, reperfusion therapy should be administered quickly to reduce infarct size and mortality (Authors/Task Force members et al., 2014). However, in NSTEMI patients, revascularization strategies are recommended based on individual clinical characteristics (Reed et al., 2017). Therefore, biomarkers with the capacity to diagnose and personalize a therapeutic schedule in AMI would be of great interest. Currently, the favored diagnostic biomarkers of AMI are cardiac troponin I (cTnI) and T (cTnT), both of which are released from necrotic cardiomyocytes within 2 to 4 h post-MI (Babuin and Jaffe, 2005), with maximum levels at 24 to 48 h and lasting for more than 1 week (Jaffe et al., 2006). For this reason, small repeat infarctions after the main infarction are difficult to detect. Thus, it is fundamental to identify biomarkers for very early diagnosis of STEMI and for monitoring the entire pathological process of AMI (**Table 3** and **Figure 3**).

**Table 3:** Epigenetic biomarkers in myocardial infarction.

Epigenetic modification	Biomarker	Regulation in myocardial infarction	Sample source	Study type	References
**DNA methylation**	*INS, GNASAS*	Hypermethylation	Leukocytes	Clinical	(Talens et al., 2012)
	*LINE-1, ZBTB12*	Hypomethylation	White blood cells	Clinical	(Guarrera et al., 2015)
	*ALDH2 promoter*	Hypermethylation	Experimental: rat model of MI	Experimental: rat model of MI	(Wang et al., 2015)
	*ZFHX3, SMARCA4*	Methylation	Whole blood	Clinical	(Nakatochi et al., 2017)
**Histone modifications**	p300	Overexpression	Myocardium	Experimental: mouse model of MI in HATmut p300-Tg mice	(Miyamoto et al., 2006)
	SUV39H, SIRT1	SUV39H upregulation and SIRT1 downregulation	H9C2 cells primary rat neonatal ventricular myocytes	Experimental: mouse model of MI in SUV39H−/− mice	(Yang et al., 2017a)
	HDAC4	Overexpression	Myocardium	Experimental: mouse model of MI in MHC-HDAC4-Tg mice	(Zhang et al., 2018b)
	HDAC6	Higher levels	Myocardium	Experimental: rat model of MI	(Nagata et al., 2019)
**miRNA**	miR-1	Higher levels	Plasma	Clinical	(Ai et al., 2010)
	miR-31, miR-126, miR-214, miR-499-5p	miR-31, miR-214: upregulation; miR-126, miR-499-5p: downregulation	Myocardium	Experimental: rat model of MI	(Shi et al., 2010)
	miR-499	Higher levels	Tissues and plasma	Clinical	(Adachi et al., 2010)
	miR-1, miR-133a, miR-133b, miR-499-5p, miR-122, miR-375	miR-1, miR-133a, miR-133b, miR-499-5p: upregulation; miR-122, miR-375: downregulation	Plasma	Clinical and experimental: mouse model of MI	(D’Alessandra et al., 2010)
	miR-1, miR-126	miR-1: increased; miR-126: decreased	Plasma	Clinical	(Long et al., 2012a)
	miR-133a	Higher levels	Plasma	Clinical	(Eitel et al., 2012)
	miR-30a, miR-195, let-7b	miR-30a, miR-195: increased; let-7: decreased	Plasma	Clinical	(Long et al., 2012b)
	miR-499-5p	Higher levels	Plasma	Clinical	(Olivieri et al., 2013)
	miR-1, miR-133a, miR-208b, miR-499	Higher levels	Plasma	Clinical	(Li et al., 2013)
	miR-150	Downregulation	plasma	Clinical	(Devaux et al., 2013)
	miR-133a	Higher levels	Plasma	Clinical	(Wang et al., 2013a)
	miR-21-5p, miR-361-5p, miR-519e-5p	miR-21-5p, miR-361-5p: increased; miR-519e-5p: reduced	Plasma	Clinical	(Wang et al., 2014b)
	miR-208a, miR-499	Higher levels in serum; miR-499: lower levels in scar, miR-208a: unchanged in scar	Serum and heart tissues	Experimental: mouse model of MI	(Xiao et al., 2014)
	miR-208b, miR-34a	Higher levels	Plasma	Clinical	(Lv et al., 2014)
	miR-328, miR-134	Higher levels	Plasma	Clinical	(He et al., 2014)
	miR-133, miR-1291, miR-663b	Higher levels	Plasma	Clinical	(Peng et al., 2014)
	miR-497	Upregulation	Plasma	Clinical	(Li et al., 2014b)
	miR-1	Higher levels	Plasma	Clinical	(Li et al., 2014a)
	miR-19a	Higher levels	Plasma	Clinical	(Zhong et al., 2014)
	miR-486-3p, miR-150-3p, miR-126-3p, miR-26a-5p, and miR-191-5p	miR-486-3p, miR-150-3p: upregulation; miR-126-3p, miR-26a-5p, miR-191-5p: downregulation	Serum	Clinical	(Hsu et al., 2014)
	miR-145	Higher levels	Serum	Clinical	(Dong et al., 2015)
	hsa-miR-493-5p, hsa-miR-369-3p, hsa-miR-495, hsa-miR-3615, hsa-miR-433, hsa-miR-877-3p, hsa-miR-1306-3p, hsv1-miR-H2, hsa-miR-3130-5p, hcmv-miR-UL22A	hsa-miR-493-5p, hsa-miR-369-3p, hsa-miR-495, hsa-miR-3615, hsa-miR-433: upregulation, hsa-miR-877-3p, hsa-miR-1306-3p, hsv1-miR-H2, hsa-miR-3130-5p, hcmv-miR-UL22A: downregulation	Plasma	Clinical	(Liang et al., 2015)
	miR-499	Higher levels	Plasma	Clinical	(Zhang et al., 2015b)
	miR-486, miR-150	Higher levels	Plasma	Clinical	(Zhang et al., 2015c)
	miR-499	Higher levels	Plasma	Clinical	(Chen et al., 2015b)
	miR-146a, miR-21	Higher levels	Plasma	Clinical	(Liu et al., 2015a)
	miR-1, miR-208, miR-499	Higher levels	Plasma	Clinical	(Liu et al., 2015b)
	miR-208a	Higher levels	Plasma	Clinical	(Białek et al., 2015)
	miR-208	Overexpression	Plasma	Clinical	(Han et al., 2015)
	miR-122-5p	Higher levels	Plasma	Clinical	(Yao et al., 2015)
	miR-21	Higher levels	Plasma	Clinical	(Zhang et al., 2016)
	miR-99a	Downregulation	Plasma	Clinical	(Yang et al., 2016a)
	miR-19b-3p, miR-134-5p and miR-186-5p	Higher levels	Plasma	Clinical	(Wang et al., 2016b)
	miR-106a-5p, miR-424-5p, let-7g-5p, miR-144-3p, miR-660-5p	Higher levels	Blood	Clinical	(Bye et al., 2016)
	miR-19b-3p, miR-134-5p and miR-186-5p	Overexpression	Plasma	Clinical	(Wang et al., 2016b)
	miR-125b-5p, miR-30d-5p	Overexpression	Plasma	Clinical	(Jia et al., 2016)
	miR-423-5p, miR-30d	Overexpression	Plasma	Clinical	(Eryılmaz et al., 2016)
	miR-221-3p	Overexpression	Plasma	Clinical	(Coskunpinar et al., 2016)
	miR-208a	Overexpression in myocardium and high levels in serum	Myocardium and serum	Experimental: rat model of MI	(Feng et al., 2016)
	miR-133b, miR-22-5p	Upregulation	Serum/plasma	Clinical	(Maciejak et al., 2016)
	miR-103a	Higher levels in plasma	Plasma and peripheral blood mononuclear cells	Clinical and experimental: *in vitro*	(Huang et al., 2016a)
	miR-122-5p/133b	High ratio	Serum	Clinical	(Cortez-Dias et al., 2016)
	miR499a-5p	Higher levels	Plasma	Clinical	(O’Sullivan et al., 2016)
	miR-181a	Higher levels	Plasma	Clinical	(Zhu et al., 2016)
	miR-145	Decreased	Plasma	Clinical	(Zhang et al., 2017b)
	miR-133a	Higher levels	Plasma	Clinical	(Yuan et al., 2016)
	miR-208b	Higher levels	Plasma	Clinical	(Liu et al., 2017)
	miR-1, miR-92a, miR-99a, miR-143, miR-223	miR-143: increased; miR-1, miR-92a, miR-99a, miR-223: decreased	Monocytes	Clinical	(Parahuleva et al., 2017)
	miR-92a	Higher levels	Plasma	Clinical	(Zhang et al., 2017c)
	miR-208b	Overexpression	Plasma	Clinical	(Zhang and Xie, 2017)
	miR-124	Higher levels	Peripheral blood	Clinical	(Guo et al., 2017)
	miR-1, miR-21, miR-29b and miR-92a	miR-1, miR-21, miR-29b: increased	Plasma	Clinical	(Grabmaier et al., 2017)
	miR-874-3p	Downregulation	Plasma	Clinical	(Yan et al., 2017)
	pmiR-126	Lower levels	Platelet	Clinical	(Li et al., 2017b)
	miR-133a	Lower levels	Serum/Plasma	Clinical	(Zhu et al., 2018)
	miR-21	Upregulation	Serum	Clinical	(Wang et al., 2017b)
	miR-4478	Higher levels	Serum	Clinical	(Gholikhani-Darbroud et al., 2017)
	miR-23b	Higher levels	Plasma	Clinical	(Zhang et al., 2018a)
	MiR-27a, miR-31, miR-1291, miR-139-5p, miR-204, miR-375	Higher levels	GEO database	High throughput	(Wu et al., 2018a)
	miR-1, miR-133a, miR-34a	Lower levels	Myocardium	Experimental: mouse model of MI	(Qipshidze Kelm et al., 2018)
	miR-19b, miR-223, miR-483-5p	Higher levels	Plasma	Clinical	(Li et al., 2019)
	miR-17-5p, miR-126-5p, miR-145-3p	Higher levels	Plasma	Clinical	(Xue et al., 2019)
	miR-150	Lower levels	Serum	Clinical	(Lin et al., 2019)
	miR-208b, miR-499	Higher levels	Plasma	Clinical	(Devaux et al., 2012)

Regarding methylation as an indicator of MI, Talens et al. (2012) investigated the association between MI and DNA methylation at six loci described to be sensitive to prenatal nutrition. As a result, the researchers demonstrated that the risk of MI in women is associated with DNA hypermethylation at *INS* and *GNASAS*-specific loci (Talens et al., 2012). Moreover, microarray analyses investigating whole-genome DNA methylation using cases from the EPICOR study and EPIC-NL cohort (Fiorito et al., 2014) identified a hypomethylated region in the zinc finger and BTB domain-containing protein 12 (*ZBTB12*) and *LINE-1*, concluding that it is possible to detect specific methylation profiles in white blood cells a few years before MI occurs. This provides a promising early biomarker of MI (Guarrera et al., 2015). Another example is the hypermethylation of the aldehyde dehydrogenase 2 gene (*ALDH2*) promoter, which is associated with myocardial injury after MI in rats. The hypermethylation downregulates *ALDH2*, inhibiting its cardioprotective role (Wang et al., 2015). Rask-Andersen et al. (2016) performed an epigenome-wide association study to identify disease-specific alterations in DNA methylation. The authors observed differential DNA methylation at 211 CpG sites in individuals with MI, and some of these sites represented genes related to cardiac function, CVD, cardiogenesis, and recovery after ischemic injury. Their results highlight genes that might be important in the pathogenesis of MI or in recovery (Rask-Andersen et al., 2016). Along the same lines, a genome-wide DNA methylation and gene ontology analysis of white blood cells from a population-based study identified four differentially methylated sites in individuals who had a previous MI. Interestingly, they found a correlation between differences in DNA methylation in blood cells and the levels of growth differentiation factor 15 (GDF-15), which was overexpressed in the myocardium of MI patients (Ek et al., 2016). Later, a genome-wide DNA methylation study of whole blood samples from MI patients and controls identified two methylated regions in zinc finger homeobox 3 (*ZFHX3*) and SWI/SNF-related, matrix-associated, actin-dependent regulator of chromatin, subfamily a, member 4 (*SMARCA4*) that were independently related to MI (Nakatochi et al., 2017).

Histone modifications are also involved in the pathological process of MI. To investigate the role of the HAT p300 in adverse left ventricular (LV) remodeling, Miyamoto et al. (2006) generated transgenic mice overexpressing wild-type p300 or its mutant in the heart. They subjected these mice to surgical MI and demonstrated that cardiac overexpression of p300 stimulated adverse LV remodeling. They concluded that the HAT activity of p300 is fundamental for the pathological course of MI (Miyamoto et al., 2006). Moreover, the class III deacetylase sirtuin 1 (SIRT1) is well known to confer a cardioprotective effect and is downregulated after cardiac injury. To understand the underlying mechanism, primary rat neonatal ventricular myocytes were exposed to ischemic or oxidative stress, leading to upregulation of the histone H3K9 methyltransferase SUV39H and downregulation of *SIRT1*. In addition, inhibition of SUV39H activity by chaetocin in wild-type mice and *SUV39H*-knockout mice protected against induced MI. SUV39H and heterochromatin protein 1 gamma cooperate to methylate the *SIRT1* promoter and repress its transcription. Thus, the authors described a role for SUV39H linking SIRT1 repression to MI (Yang et al., 2017a). To examine the role of HDAC4 in the modulation of cardiac function after an MI, Zhang et al. (2018b) generated a myocyte-specific activated HDAC4-transgenic mouse. They found that HDAC4 overexpression increases myocardial fibrosis and hypertrophy, leading to cardiac dysfunction. Furthermore, the overexpression of activated HDAC4 aggravated cardiac dysfunction and increased adverse remodeling and apoptosis in the infarcted myocardium. Thus, HDAC4 is an indicator of heart injury (Zhang et al., 2018b). More recently, the role of HDAC6 in the development of HF following MI was investigated using a rat model. The authors found that the deacetylase activity of HDAC6 is increased after MI (Nagata et al., 2019).

Abundant research has focused on miRNAs as novel biomarkers for MI. MiR-1 levels have been analyzed in plasma from patients with AMI and found to be significantly elevated, but decreased to normal levels with medication (Ai et al., 2010; Long et al., 2012a). MiR-1, miR-126, and cTnI expression levels exhibited a similar tendency. Thus, circulating miR-1 and miR-126 may be useful indicators of AMI (Long et al., 2012a). However, when miR-1 was compared to cTnT, the authors found that cTnT was more specific and sensitive than miR-1 (Li et al., 2014a). Experiments performed in a rat model of MI revealed dysregulation of several miRNAs in the myocardium. Specifically, miR-31, miR-208, and miR-214 were upregulated, and miR-126 and miR-499-5p were downregulated in infarcted rats compared to sham-operated animals (Ji et al., 2009; Shi et al., 2010). MiR-499 has been widely analyzed as a possible biomarker of MI. MiR-499 has been reported to be produced almost exclusively in the heart and plasma and is significantly increased in individuals with AMI (Adachi et al., 2010; Devaux et al., 2012). MiR-499 positively correlates with serum creatine kinase-MB (CK-MB) and cTnI increasing their diagnostic accuracy (Chen et al., 2015b; Zhang et al., 2015b). Thus, miR-499 might be a suitable biomarker for MI and a predictor of myocardial ischemia risk (Adachi et al., 2010; Chen et al., 2015b; Zhang et al., 2015b). These results were confirmed in the mouse model of MI, with elevated serum miR-208a levels. However, the expression of miR-499 was significantly reduced in the MI region, whereas miR-208a remained unchanged in the same area. One explanation is that the damaged heart might release miR-499 into the circulation (Xiao et al., 2014). Other authors observed a high correlation between circulating miRNA-208a in STEMI patients and the levels of cTnI and CK-MB mass liberated from the infarcted zone (Białek et al., 2015). Thus, cardiac miR-208 and miR-499 seemed to be better biomarkers for predicting AMI than miR-1 (Liu et al., 2015b; Liu et al., 2018a). Another study analyzed the expression of miR-208a in the myocardium and serum of infarcted rats compared to control groups, as well as the expression of cAMP-PKA to evaluate the effect of this signaling pathway in the primary stages of MI; they found increased expression of miR-208a and cAMP-PKA. Moreover, the transfection of human myocardial cells with the miR-208a analog significantly increased the amount of cAMP-PKA protein. Thus, higher expression of miR-208a in the infarcted myocardium and serum may play a role in MI by affecting the cAMP-PKA signaling pathway (Feng et al., 2016).

D’Alessandra et al. (2010) investigated plasma levels of miRNAs in acute STEMI patients and infarcted mice and found higher levels of miR-1, miR-133a, miR-133b, and miR-499-5p compared to controls, whereas miR-122 and miR-375 levels were lower only in STEMI patients. Peak miR-1, miR-133a, and miR-133b expression correlated with cTnI levels in time, whereas the time course of miR-499-5p was slower (D’Alessandra et al., 2010). This was later confirmed in an exhaustive meta-analysis of relevant publications (Cheng et al., 2014). Similarly, geriatric patients with acute NSTEMI had greater miR-499-5p levels, exhibiting greater precision in diagnosis than cTnT in patients with mild ST elevation (Olivieri et al., 2013). On the other hand, increased levels of miR-1, miR-133a, miR-208b, and miR-499 in patients with AMI have been demonstrated to not be superior to cTnT (Li et al., 2013). The use of miR-133a as a biomarker in reperfused STEMI has been evaluated and compared to cardiovascular magnetic resonance imaging; high levels of miR-133a correlated with an increased infarct scar size, worse myocardial recovery, and prominent reperfusion injury. Nevertheless, miR-133a did not add further predictive information to cardiovascular magnetic resonance and conventional markers used in clinical practice in high-risk STEMI patients (Eitel et al., 2012). Moreover, the circulating levels of miR-133a were significantly enhanced in AMI patients compared to coronary heart disease and myocardial ischemia patients, presenting a similar trend as plasma cTnI concentration. Remarkably, we found a positive correlation between circulating miR-133a levels and the severity of coronary artery stenosis. Thus, circulating miR-133a may be a suitable tool for AMI diagnosis and predicting the presence and severity of coronary damage in coronary heart disease patients (Wang et al., 2013a). These results were later confirmed (Yuan et al., 2016; Zhu et al., 2018). Nevertheless, in another study analyzing miR-133a and miR-423-5p and their relationship with cardiac biomarkers, such as B-type natriuretic peptide (BNP), C-reactive protein, and cTnI in MI patients, an increase in circulating levels of both miRNAs was observed, but these changes were not associated with LV remodeling or BNP. The authors claimed that miR-133a and miR-423-5p are not useful biomarkers of LV remodeling after MI (Bauters et al., 2013). Another controversial pair of biomarkers is miR-423-5p and miR-30d, which were found to be higher in STEMI patients without a significant correlation with cTnI (Eryılmaz et al., 2016). In addition, the analysis of circulating miR-124a and miR-133 in STEMI and cardiogenic shock patients revealed a significant upregulation of both molecules. A negative correlation was found between miR-133 and MMP-9 levels, and a relationship between miR-124 and soluble ST2 levels, a marker associated with cardiac damage. Surprisingly, this study did not connect any of the miRNAs to the extent of the injury, disease progression, or the prognosis of patient outcomes. In this case, miRNAs would not bring any benefit compared to current markers (Goldbergova et al., 2018). Moreover, elevated circulating miR-1254 was described as predicting adverse LV remodeling in STEMI patients when compared to magnetic resonance imaging. However, the diagnosis and prognosis values of miR-1254 require further research (de Gonzalo-Calvo et al., 2018). Other investigations have described miR-150-3p and miR-486-3p as being upregulated, whereas miR-26a-5p, miR-126-3p, and miR-191-5p were significantly downregulated in STEMI patients (Hsu et al., 2014). In the same manner, circulating miR-19b-3p, miR-134-5p, and miR-186-5p have been reported to be significantly elevated in the initial stages of AMI. The expression of miR-19b-3p and miR-134-5p in the plasma reached a maximum earlier than miR-186-5p. However, all three positively correlated with cTnI and achieved peak expression before cTnI, which was 8 h after admission. Interestingly, the expression of these circulating miRNAs was not altered by heparin and medications for AMI, and the combination of all three miRNAs increased their diagnostic efficacy (Wang et al., 2016b). Moreover, a higher miR-122-5p/133b ratio was found in serum from STEMI patients (Cortez-Dias et al., 2016). The NSTEMI patients presented higher serum levels of miR-4478, soluble leptin receptor, cTnI, CKMB, urea, creatinine, glucose, cholesterol, TG, and ALP but lower levels of ALT compared to healthy individuals (Gholikhani-Darbroud et al., 2017). Moreover, there was an increase in miR-143 expression in monocytes from STEMI patients, whereas miR-1, miR-92a, miR-99a, and miR-223 expression was significantly reduced. Also, monocytic expression of miR-143 positively correlated with high-sensitivity C-reactive protein (hs-CRP), but not cTnT. These findings demonstrated that circulating monocytes could also be suitable biomarkers (Parahuleva et al., 2017).

Interestingly, cell-specific miRNA patterns are able to distinguish STEMI and NSTEMI patients. A correlation was found between miRNA 30d-5p and plasma, platelets, and leukocytes in patients with STEMI and NSTEMI. Furthermore, miR-221-3p and miR-483-5p were associated with plasma and platelets, but only in NSTEMI patients (Ward et al., 2013).

High levels of plasma miR-134 and miR-328 are described as being possible AMI biomarkers, as they correlate with a superior risk of developing HF and mortality. However, the miRNA levels were not superior to high-sensitivity cTnT (hs-cTnT) concentrations (He et al., 2014). In addition, elevated levels of miR-19a, miR-22-5p, miR-27a, miR-30a, miR-30a-5p, miR-30d-5p, miR-31, miR-34a, miR-122-5p, miR-125b-5p, miR-133, miR-133b, miR-139-5p, miR-150, miR-181a, miR-195, miR-204, miR-208, miR-208b, miR-221-3p, miR-375, miR-486, miR-497, miR-499a-5p, miR-663b, miR-1291, and let-7b can be potential biomarkers for AMI, increased risk of mortality, or HF (Devaux et al., 2012; Long et al., 2012b; Devaux et al., 2013; Li et al., 2014b; Lv et al., 2014; Peng et al., 2014; Zhong et al., 2014; Han et al., 2015; Yao et al., 2015; Zhang et al., 2015c; Coskunpinar et al., 2016; Jia et al., 2016; Maciejak et al., 2016; O’Sullivan et al., 2016; Zhu et al., 2016; Liu et al., 2017; Zhang and Xie, 2017; Alavi-Moghaddam et al., 2018; Maciejak et al., 2018; Wu et al., 2018a; Wang et al., 2019b). Other potential biomarkers for AMI are downregulated in patients’ plasma, such as miR-99a, miR-122-5p, and miR-874-3p (Yang et al., 2016a; Yan et al., 2017; Wang et al., 2019b). Interestingly, high levels of the combination of miR-21-5p, miR-361-5p, and miR-519e-5p or the reduction of miR-519e-5p correlates with cTnI concentrations, significantly increasing the diagnostic accuracy in AMI patients (Wang et al., 2014b;Liu et al., 2015a). Similarly, miR-21 and miR-124 have similar diagnostic ability compared to CK, CK-MB, and cTnI (Zhang et al., 2016; Guo et al., 2017).

In an attempt to predict HF and cardiovascular death after AMI, circulating miR-145, the N-terminal fragment of the precursor BNP, myocardial-band CK, and cTnI concentrations were analyzed for short- and long-term clinical outcomes. As a result, the authors concluded that miR-145 was a significant independent predictor of cardiac events, predicting long-term outcomes after AMI (Dong et al., 2015). Later, another group found that miR-145 levels were significantly lower in AMI patients and correlate with increased serum BNP and cTnT and decreased LV ejection fraction (Zhang et al., 2017b).

An miRNA array revealed differences in the miRNA expression patterns in patients with different phases of HF after MI. Specifically, human miR-369-3p, miR-433, miR-493-5p, miR-495, and miR-3615 were overexpressed, whereas miR-877-3p, miR-1306-3p, hsv1-miR-H2, miR-3130-5p, and hcmv-miR-UL22A were underexpressed in these patients. Thus, these circulating miRNAs are novel candidates as biomarkers of MI and HF (Liang et al., 2015).

An important aspect of circulating miRNAs as biomarkers is their temporal release, source, and transportation. Using the ischemia–reperfusion injury model, Deddens et al. (2016) showed that the ischemic myocardium releases extracellular vesicles. They also demonstrated that these extracellular vesicles transported specific miRNAs from the heart and muscle and were quickly detected in plasma. Interestingly, these vesicles had a high miRNAs content and rapid detection compared to traditional injury markers. This makes them a promising tool for the early detection of MI (Deddens et al., 2016). Along the same lines, microparticles and the expression levels of miR-92a were investigated in AMI and stable coronary artery disease patients and compared to cTnI. The number of microparticles and expression levels of miR-92a were higher in AMI patients than in the stable coronary artery disease patients and control groups, with a positive correlation between the levels of microparticles and cTnI. Thus, microparticles containing miR-92a may be suitable for MI diagnosis and possibly regulate dysfunctional endothelial tissue in AMI patients (Zhang et al., 2017c). However, according to Grabmaier et al. (2017), miR-92a seems to not be a good biomarker of adverse ventricular remodeling in post-AMI patients. The authors evaluated circulating miR-1, miR-21, miR-29b, and miR-92a from the SITAGRAMI trial population and found that miR-1, miR-21, and miR-29b expression was higher in AMI patients. The levels of miR-1 and miR-29b in plasma post-AMI correlated with variations in infarct volume, and the levels of miR-29b and changes in LV ejection fraction over time were also associated (Grabmaier et al., 2017).

Investigation of the expression of miR-103a in AMI patients with and without high blood pressure and the effect on endothelial cell function revealed increased levels of miR-103a in all patients but no changes in peripheral blood mononuclear cells. Moreover, miR-103a suppressed the expression of Piezo1 protein, which diminished the capacity to produce capillary tubes and the viability of human umbilical vein endothelial cells (HUVECs). Thus, miR-103a may take part in the development of high blood pressure and the initiation of AMI *via* regulation of Piezo1 expression (Huang et al., 2016a).

In a study based on samples from the HUNT study biobank, Bye et al. (2016) analyzed the utility of circulating miRNAs to predict future fatal AMI in healthy participants. MiR-424-5p and miR-26a-5p were associated exclusively with risk in men and women, respectively, suggesting a gender-specific association. They discovered that the best model for predicting future AMI consisted of miR-106a-5p, miR-424-5p, let-7g-5p, miR-144-3p, and miR-660-5p, and these miRNAs were proposed as a panel to enhance the prediction of AMI risk in healthy individuals (Bye et al., 2016).

Platelet activation is critical for AMI pathogenesis, but the role of platelet miRNAs (pmiRNAs) as biomarkers in AMI and their correlation with indices of platelet activity are unclear. Assessment of pmiR-126 expression in STEMI patients revealed reduced levels and a correlation with plasma cTnI. However, pmiR-126 expression did not correlate well with platelet activity indices, and its potential diagnostic utility is limited (Li et al., 2017b).

MiR-1, miR-133a, and miR-34a induce adverse structural remodeling to impair cardiac contractile function. Increased levels of all three miRNAs have been shown in the hearts of old MI mice compared to young MI mice, and the miR-1 increase was more prolonged and corresponded to LV wall thinning. This suggests that significantly increased levels of miR-1 in the aged post-MI heart could be a biomarker for high-risk prediction (Qipshidze Kelm et al., 2018). In addition, miRNA-21 has been reported to be overexpressed in the serum of ancient patients with AMI and to positively correlate with serum levels of CK-MB and cTnI. *In vitro* experiments with human cardiomyocytes transfected with the miR-21 mimic short hairpin RNA have shown that, following tumor necrosis factor α (TNF-α) induction, apoptosis rates are downregulated. The upregulation of miR-21 expression in the serum of elderly patients with AMI inhibited apoptosis induced by TNF-α in human cardiomyocytes *via* activation of the JNK/p38/caspase-3 signaling pathway (Wang et al., 2017b). Along the same lines, cardiomyocyte apoptosis and hypoxic reduction of cell growth can be promoted by miR-23b overexpression, suggesting that it could be a potential biomarker for STEMI (Zhang et al., 2018a).

A recent study explored the diagnostic use of circulating miRNAs in patients with acute chest pain in the emergency department. They found that higher circulating miR-19b, miR-223, and miR-483-5p levels may be clinically useful for AMI diagnosis in early phases (Li et al., 2019). Similarly, circulating miR-17-5p, miR-126-5p, and miR-145-3p levels are elevated in plasma from AMI patients. Combining these three miRNAs achieves a more precise AMI diagnosis (Xue et al., 2019). Interestingly, next-generation miRNA sequencing from whole blood samples has been useful for identifying new biomarkers of MI (Kanuri et al., 2018).

### Heart Failure

Heart failure is a chronic and progressive condition that hampers the ability of the heart to pump enough blood to the body and fulfill its needs. Heart failure is caused by multiple disorders, such as hypertension, cardiomyopathy, MI, arrhythmias, or valvular diseases, among others (Khatibzadeh et al., 2013). Numerous scientific reports connect HF and epigenetic modifications (**Table 4** and **Figure 3**). High-density epigenome-wide mapping of DNA methylation in the myocardium and blood from dilated cardiomyopathy patients and healthy individuals has been analyzed. This technology has been used to find regions of epigenetic susceptibility and new biomarkers related to HF and heart dysfunction; they recognized different patterns of epigenetic methylation that were preserved through tissues—the CpGs regions identified as novel biomarkers of HF (Meder et al., 2017; Rau and Vondriska, 2017). Differentially methylated DNA regions were also identified in blood leukocytes from HF patients (Li et al., 2017a). Dilated cardiomyopathy is an important cause of HF. Genome-wide cardiac DNA methylation in idiopathic dilated cardiomyopathy patients revealed abnormal DNA methylation, which was related to important variations in the expression of lymphocyte antigen 75 (*LY75*) and adenosine receptor A2A (*ADORA2A*) mRNA (Haas et al., 2013). Similarly, genome-wide maps of DNA methylation and enrichment of histone 3 lysine-36 trimethylation (H3K36me3) in pathological and healthy hearts were analyzed. Differences in DNA methylation were found in promoter CpG islands, genes, intragenic CpG islands, and H3K36me3-rich regions of the genome. The promoters of upregulated genes had altered DNA methylation, but not the promoters of downregulated genes. In particular, an abundance of *DUX4* transcripts was associated with differences in DNA methylation and H3K36me3 enrichment. Although further studies need to be carried out, there is evidence that the expression of genes critical for the development of cardiomyopathies may be controlled by the epigenome (Movassagh et al., 2011). Moreover, in patients with dilated cardiomyopathy, there is an altered methylation pattern in the regulatory regions of cardiac development genes, such as T-box protein 5 (*TBX5*), heart and neural crest derivatives expressed 1 (*HAND1*), and NK2 homeobox 5 (*NKX2.5*) (Jo et al., 2016). Koczor et al. (2013) also studied the differential methylation patterns in patients with dilated cardiomyopathy, which is characterized by congestive HF. Computational analysis detected few differentially methylated gene promoters (*AURKB*, *BTNL9*, *CLDN5*, and *TK1*). This study provides relevant information on DNA methylation and altered expression in dilated cardiomyopathy that would help in treatment (Koczor et al., 2013).

**Table 4:** Epigenetic biomarkers in heart failure.

Epigenetic modification	Biomarker	Regulation in heart failure	Sample source	Study type	References
**DNA methylation**	*LY75* and *ADORA2A*	Aberrant DNA methylation	Left ventricle myocardium and zebrafish	Clinical and experimental: zebrafish	(Haas et al., 2013)
	*TBX5*, *HAND1*, and *NKX2.5*	Altered DNA methylation	Myocardium	Clinical	(Jo et al., 2016)
	*HEY2*, *MSR1*, *MYOM3*, *COX17*, miR-24-1, *CTGF*, *MMP2*, miR-155	*HEY2*, *MSR1*, *MYOM3*, *COX17*, and miR-24-1: hypermethylation; *CTGF*, *MMP2*, and miR-155: hypomethylation	Myocardium	Clinical	(Glezeva et al., 2019)
**Histone modifications**	H3K36me3	H3K36me3 enhancement	Myocardium	Clinical	(Movassagh et al., 2011)
	HDAC4	HDAC4 activation	Myocardium	Clinical and experimental: mouse model of pressure overload	(Hohl et al., 2013)
	H3K4me2, H3K9me2, H3K27me3, H3K36me2, KDM2A	H3K4me2, H3K9me2, H3K27me3, and H3K36me2 methylation and KDM2A reduction	Myocardium	Experimental: mouse model of pressure overload	(Angrisano et al., 2014)
	Atp2a2	*Atp2a2* acetylation	Ventricular myocytes and myocardium	Clinical and experimental: mouse model of pressure overload in *MHC-SIRT1*−/− Tg mice and swine model of MI	(Gorski et al., 2019)
**miRNA**	miR423-5p	Higher levels	Plasma	Clinical	(Tijsen et al., 2010)
	miR-192	Upregulation	Serum	Clinical	(Matsumoto et al., 2013)
	miR-122*, miR-200b, miR-520d-5p, miR-622, miR-1228*, miR-558	miR-122*, miR-200b, miR-520d-5p, miR-622, miR-1228*: upregulation; miR-558: downregulation	Whole peripheral blood	Clinical	(Vogel et al., 2013)
	miR-103, miR-142-3p, miR-30b, miR-342-3p	Differentially expressed	Plasma	Clinical	(Ellis et al., 2013)
	miR-210, miR-30a	Upregulation	Serum	Clinical	(Zhao et al., 2013)
	miR-210	Higher levels	Plasma, mononuclear cells, and skeletal muscles	Clinical and experimental: Dahl salt-sensitive rats	(Endo et al., 2013)
	miR-1	Higher levels	Plasma	Clinical	(Zhang et al., 2013b)
	miR-423-5p	Positive transcoronary gradients	Transcoronary gradients	Clinical	(Goldraich et al., 2014)
	miR-423-5p	Lower levels	Plasma	Clinical	(Seronde et al., 2015)
	MiR-30c, miR-146a, miR-221, miR-328, miR-375	Downregulation	Serum	Clinical	(Watson et al., 2015)
	miR-21, miR-650, miR-744, miR-516-5p, miR-1292, miR-182, miR-1228, miR-595, miR-663b, miR-1296, miR-1825, miR-299-3p, miR-662 miR-122, miR-3148, miR-518e, miR-129-3p, miR-3155, miR-3175, miR-583, miR-568, miR-30d, miR-200a-star, miR-1979, miR-371-3p, miR-155-star, miR-502-5p	miR-21, miR-650, miR-744, miR-516-5p, miR-1292, miR-182, miR-1228, miR-595, miR-663b, miR-1296, miR-1825, miR-299-3p, miR-662 miR-122, miR-3148, miR-518e: increased; miR-129-3p, miR-3155, miR-3175, miR-583, miR-568, miR-30d, miR-200a-star, miR-1979, miR-371-3p, miR-155-star, miR-502-5p: decreased	Serum	Clinical	(Cakmak et al., 2015)
	miR-1233, miR-671-5p, miR-183-3p, miR-190a, miR-193b-3p, miR-193b-5p, miR-211-5p, miR-494	miR-1233, miR-671-5p: Upregulation; miR-183-3p, miR-190a, miR-193b-3p, miR-193b-5p, miR-211-5p, miR-494: downregulation	Whole blood and plasma	Clinical	(Wong et al., 2015)
	miR-1, miR-21	miR-1: downregulation; miR-21: upregulation	Serum	Clinical	(Sygitowicz et al., 2015)
	miR-126	Downregulation	Serum	Clinical	(Wei et al., 2015)
	miR-1, miR-21, miR-23, miR-29, miR-130, miR-195, miR-199	Upregulation	Myocardial biopsy	Clinical	(Lai et al., 2015)
	miR-106a-5p, miR-223-3p, miR-652-3p, miR-199a-3p, miR-18a-5p	Downregulation	Plasma	Clinical	(Vegter et al., 2016)
	miR-148b-3p, miR-409-3p	Downregulation	Serum and left atrial tissue	Clinical	(Chen et al., 2016)
	miR-122-5p, miR-184	Upregulation	H9C2 cells and blood and myocardium	Experimental: *in vitro* and rat model of post-MI HF	(Liu et al., 2016)
	miR-660-3p, miR-665, miR-1285-3p, miR-4491	Upregulation	Plasma and heart	Clinical	(Li et al., 2016)
	miR-18a-5p, miR-26b-5p, miR-27a-3p, miR-30e-5p, miR-106a-5p, miR-199a-3p, miR-652-3p	Lower levels	Plasma	Clinical	(Ovchinnikova et al., 2016)
	miR-19b	Lower levels	Serum and myocardial	Clinical and experimental: *in vitro*	(Beaumont et al., 2017)
	miR-30d	Lower levels	Serum	Clinical	(Xiao et al., 2017)
	miR-195-3p	Higher levels	Plasma	Clinical	(He et al., 2017)
	miR-22-3p	Higher levels	Blood	Clinical	(van Boven et al., 2017)
	miR-150-5p	Downregulation	Blood	Clinical	(Scrutinio et al., 2017)
	miR-133b-3p, miR-208b-3p, miR-125a-5p, miR-125b-5p, miR-126-3p, miR-21-5p, miR-210-3p, miR-29a-3p, miR-320a, miR-494-3p	Upregulation	Blood	Experimental: sheep model of HF	(Wong et al., 2017)
	miR-146a	Upregulation	Exosomal and total plasma	Clinical and experimental: *in vitro*	(Beg et al., 2017)
	miR-9, miR-495, miR-599, miR-181c	ex-miR-9, ex-miR-181c, ex-miR-495: increased; ex-miR-599: decreased	Exosomal and total plasma	Experimental: dogs with myxomatous mitral valve disease, mitral valve prolapse	(Yang et al., 2017b)
	miR-21-5p, miR-23a-3p, miR-222-3p	Higher levels	Plasma	Clinical and experimental: rat model of post-MI HF	(Dubois-Deruy et al., 2017)
	miRNA-21	Higher levels	Serum	Clinical	(Zhang et al., 2017a)
	miR-132	Higher levels	Plasma	Clinical	(Masson et al., 2018)
	miR-1254, miR-1306-5p	Higher levels	Blood	Clinical	(Bayés-Genis et al., 2018)
	miR-423, miR-34a, miR-21-3p, miR-30a	miR-21-3p, miR-30a: Positive transcoronary gradient in non-ischemic HF; miR-423, miR-34a: Negative transcoronary gradient in ischemic HF	Transcoronary gradients	Clinical	(De Rosa et al., 2018)
	miR-3135b, miR-3908, miR-5571-5p	Upregulation	Plasma	Clinical	(Chen et al., 2018a)
	miR-302b-3p	Higher levels	Plasma	Clinical	(Li et al., 2018a)
	exo-miR-92b-5p	increased	Serum	Clinical	(Wu et al., 2018c)
	miR-26b, miR-208b, miR-499	Higher levels	Peripheral blood mononuclear cells	Clinical	(Marketou et al., 2018)
	miR-423-5p, miR-221-5p, miR-212-5p, miR-193b-5p, miR-15a-5p, miR-208a-3p	Upregulation	Plasma, mouse myocardium and NRVMs cells	Clinical and experimental: *in vitro*, and murine model of hypertrophy and HF	(Shah et al., 2018b)
	miR-192	Upregulation	Serum	Clinical	(Klenke et al., 2018)
	miR-34a, miR-208b, miR-126, miR-24, miR-29a	miR-34a, miR-208b, miR-126: upregulation; miR-24, miR-29a: downregulation	Serum	Clinical	(Lakhani et al., 2018)
	miR-17, miR-20a, miR-106b	Lower levels	Plasma	Clinical	(Shah et al., 2018a)
	miR-197-5p	Upregulation	Plasma	Clinical	(Liu et al., 2018b)
	miR-133a, miR-221	Higher levels	Plasma	Clinical	(Guo et al., 2018)
	exo-miR-92b-5p	Higher levels	Serum	Clinical	(Wu et al., 2018b; Wu et al., 2018c)

Furthermore, epigenetic modifications have been proposed to play an important role in HF progression in the murine model of pressure overload. The researchers observed a reduction in sarcoplasmic reticulum Ca^2+^ATPase (*Atp2a2*) levels and a significant induction of β-myosin-heavy chain (*Myh7*) mRNA levels. They also detected H3K4me2, H3K9me2, H3K27me3, and H3K36me2 and a reduction in the lysine-specific demethylase KDM2A after 8 weeks of transverse aortic constriction (Angrisano et al., 2014). *Atp2a2* is a determinant of cardiac function, and its reduced activity is a clear feature of HF. Gorski et al. (2019) investigated the role of lysine acetylation in *Atp2a2* function in HF patients and found that acetylation at lysine 492 is regulated by SIRT1 and HAT p300 and significantly reduced the gene activity (Gorski et al., 2019). All of this knowledge would be fundamental to identifying potential biomarkers and new epigenetic drugs in HF therapy. Interestingly, an association has been reported between epigenetic remodeling in the atrial natriuretic peptide (*ANP*) and *BNP* promoters and reactivation of the fetal gene program in HF. Their reported upregulation in HF patients did not respond to an increase in histone acetylation but HDAC4, which is exported from the nucleus. In contrast, demethylation of H3K9 and dissociation of heterochromatin protein 1 from gene promoters were regulated by HDAC4. Thus, HDAC4 is fundamental to histone methylation in HF caused by increased cardiac load and a potential target for treatment (Hohl et al., 2013). More recently, Glezeva et al. (2019) performed targeted DNA methylation sequencing to detect DNA methylation alterations in coding and ncRNA in cardiac interventricular septal tissue from HF patients. They found hypermethylation in *HEY2*, *MSR1*, *MYOM3*, *COX17*, and miR-24-1 and hypomethylation in *CTGF*, *MMP2*, and miR-155. Therefore, they defended a unique cohort of loci useful as diagnostic and therapeutic targets in HF (Glezeva et al., 2019).

More than 10 years ago, few reports suggested that specific miRNAs are differentially regulated in the failing heart (Divakaran and Mann, 2008; Small and Olson, 2011). Since then, an extensive evidence base has been published in the literature regarding the use of miRNAs as possible biomarkers for HF diagnosis and prognosis. In evaluating whether miRNAs can differentiate clinical HF from healthy individuals and from non-HF dyspnea, miRNA arrays have revealed miR423-5p enrichment in the blood of HF patients (Tijsen et al., 2010). However, criticisms have been raised in this study regarding age differences between groups, reduced sample size, and statistics (Kumarswamy et al., 2010). Moreover, patients with HF of different etiologies presented with different expression levels of circulating miRNAs. Ischemic HF patients were found to have a positive transcoronary gradient for miR-423-5p, miR-423, and miR-34a, but the nonischemic HF group was positive only for miR-21-3p and miR-30a. The transcoronary concentration gradient suggests that the failing heart may selectively release the miRNAs into the coronary circulation. These miRNAs could be useful for discriminating different etiologies of HF (Goldraich et al., 2014; De Rosa et al., 2018).

Circulating miRNAs have been screened in an attempt to identify any that could be used for the prognosis of ischemic HF in post-AMI patients. Knowing that p53 has been involved in HF development in mice (Sano et al., 2007), the authors took great interest in p53-responsive miRNAs. The serum levels of miR-34a, miR-192, and miR-194 were significantly and coordinately upregulated in AMI patients with ischemic HF progression, and all three were p53-responsive. Interestingly, these miRNAs were contained in extracellular vesicles, suggesting that they are circulating regulators of HF. Furthermore, there was a significant correlation between the LV end-diastolic dimension 1 year after AMI and the miR-194 and miR-34a expression levels. Thus, although further investigations are needed, these results suggest the usefulness of miR-34a, miR-192, and miR-194 in predicting the risk of HF progression after AMI (Evans and Mann, 2013; Matsumoto et al., 2013; Klenke et al., 2018).

Vogel et al. (2013) assessed the genome-wide miRNA expression profiles in HF patients with reduced ejection fraction (HFrEF). They demonstrated that dysregulated levels of miRNAs, such as miR-122*, miR-200b, miR-520d-5p, miR-622, miR-1228* (upregulated), or miR-558 (downregulated) significantly correlate with disease severity, as indicated by LV ejection fraction (Vogel et al., 2013). Moreover, Ellis et al. (2013) tried to find differences between HF patients and non–HF-related breathlessness, and between HFrEF and HF with preserved ejection fraction (HFpEF); although they found a differential expression of miR-103, miR-142-3p, miR-30b, and miR-342-3p in HF and breathless patients, individually, classical biomarkers such as NT-proBNP and hs-cTnT exhibited greater sensitivity and specificity. However, the combination of miRNAs with NT-proBNP significantly improved prediction performance (Ellis et al., 2013). Similarly, elevated plasma levels of miR-210 were reported in congestive HF patients, although no significant correlation was observed with BNP. However, patients with an improved BNP profile presented with low plasma miR-210 levels. MiR-210 might reflect a mismatch between heart contraction and oxygen demand in the peripheral tissues (Endo et al., 2013). Interestingly, miR-210 and miR-30a expression is upregulated in HF patients, with a tendency toward fetal values (Zhao et al., 2013). Moreover, changes in myocardial miRNA in patients with stable and end-stage HF partially resemble the fetal myocardium. Target mRNA levels negatively correlate with changes in highly expressed miRNAs in HF and fetal hearts. The circulation is dominated by miRNAs, fragments of tRNAs, and small cytoplasmic RNAs. Heart- and muscle-specific circulating miRNAs (myomirs) are also increased in advanced HF, correlating with cTnI levels. These findings support miRNA-based therapies and the use of circulating miRNAs as biomarkers for heart injury (Akat et al., 2014). Cardiac fibroblast–derived miRNAs, such as miR-660-3p, miR-665, miR-1285-3p, and miR-4491, have also been found to be significantly upregulated in heart and plasma during HF, discriminating patients from controls (Li et al., 2016). However, miRNAs in the pericardial fluid are not related to cardiovascular pathologies or clinically assessed stages of HF. MicroRNAs may be paracrine signaling factors that intervene in cardiac cells crosstalk (Kuosmanen et al., 2015).

In another study performed in patients with chronic congestive HF, microarray profiling demonstrated increased expression of miR-21, miR-122, miR-182, miR-299-3p, miR-516-5p, miR-518e, miR-595, miR-650, miR-662, miR-663b, miR-744, miR-1228, miR-1292, miR-1296, miR-1825, and miR-3148 and decreased expression of miR-30d, miR-129-3p, and miR-502-5p, miR-155-star miR-200a-star, miR-371-3p, miR-583, miR-568, miR-1979, miR-3155, and miR-3175. Among these miRNAs, miR-182 seemed to have a better prognostic value than hs-CRP (Cakmak et al., 2015). Furthermore, miR-30c, miR-146a, miR-221, miR-328, and miR-375 had different expression levels in HFrEF and HFpEF. The combination of two or more miRNAs with BNP could significantly improve the discrimination of these pathological conditions compared to BNP alone (Watson et al., 2015). Additional miRNAs have been identified as promising biomarkers to discriminate HF from healthy individuals and to differentiate HFrEF from HFpEF: miR-125a-5p, miR-183-3p, miR-190a, miR-193b-3p, miR-193b-5p, miR-211-5p, miR-494, miR-545-5p, miR-550a-5p, miR-638, miR-671-5p, miR-1233, miR-3135b, miR-3908, and miR-5571-5p. The use of a combination of miRNAs and NT-proBNP increases its discernment capacity (Schulte et al., 2015; Wong et al., 2015; Chen et al., 2018a). Similarly, increased levels of miR-133a and miR-221 can be used as suitable HF diagnostic biomarkers in elderly HF patients, and the combination of NT-proBNP and miR-133a can improve the diagnostic accuracy (Guo et al., 2018). Serum levels of miR-1, miR-21, and miR-208a have also been analyzed in symptomatic HF patients. Expression of miR-1 is reduced in symptomatic HF patients, with decreasing levels correlating with increasing severity. In contrast, miR-21 has been shown to be overexpressed with no relation to HF severity. No circulating miR-208a has been observed in symptomatic HF patients. A negative correlation between miR-1 expression and NT-proBNP has been reported in HF patients, whereas miR-21 and galectin-3 have been positively correlated. Therefore, dysregulated levels of miR-1 and miR-21 may be fundamental for HF progression (Sygitowicz et al., 2015). An inverse correlation between miR-1 levels and ejection fraction has also been reported. Thus, elevated levels of miR-1 may inhibit cardiac function and be a predictor of the onset of HF secondary to AMI (Zhang et al., 2013b).

MiR-126 has also been studied in atrial fibrillation and/or HF patients, with downregulated expression in patients and positive correlation with LV ejection fraction but a negative association with the cardiothoracic ratio and NT-proBNP. Thus, the reduction in miR-126 expression is a potential indicator of severity in atrial fibrillation and HF (Wei et al., 2015). A significant negative correlation has also been found between several miRNAs and classical clinical biomarkers indicative of a worse clinical outcome in HF patients. MiR-16-5p has been correlated to CRP, miR-106a-5p to creatinine, miR-223-3p to growth differentiation factor 15, miR-652-3p to soluble ST-2, miR-199a-3p to procalcitonin and galectin-3, and miR-18a-5p to procalcitonin (Vegter et al., 2016). Furthermore, an analysis of myocyte and fibroblast-related miRNAs and mRNAs in myocardium samples from HF patients and control individuals revealed that miR-1, miR-21, miR-23, miR-29, miR-130, miR-195, and miR-199 are significantly upregulated in HF patients, whereas miR-30, miR-133, miR-208, and miR-320 do not significantly change. Related mRNAs, such as caspase 3, collagenase I, collagenase III, and transforming growth factor (TGF), are also upregulated in HF patients. MicroRNAs involved in apoptosis, hypertrophy, and fibrosis are upregulated in the myocardium of HF patients and may be suitable biomarkers in the early stages of chronic HF and future therapeutic targets (Lai et al., 2015).

Evaluation of miR-148b-3p and miR-409-3p in mitral regurgitation patients, asymptomatic mitral regurgitation patients, and controls revealed that circulating and tissue miR-148b-3p and circulating miR-409-3p are significantly downregulated in mitral regurgitation patients with HF, and miR-148b-3p is significantly downregulated only in the mitral regurgitation patients without HF. Notably, the mRNAs of target genes of both miRNAs have been shown to be upregulated in HF patients with mitral regurgitation. Thus, circulating miR-148b-3p may be used as a biomarker of HF and miR-409-3p for incident HF in mitral regurgitation patients (Chen et al., 2016).

Specific overexpression of miR-221 in the hearts of transgenic mice has been shown to induce cardiac dysfunction and HF by impairing autophagy. In addition, *in vitro* miR-221 upregulation inhibits autophagic vesicle formation. Thus, autophagy balance and cardiac remodeling are regulated by miR-221 levels through modulation of the p27/CDK2/mTOR axis, and miR-221 might be a therapeutic target in HF (Su et al., 2015). Furthermore, high-throughput sequencing has been used to determine the differential miRNA pattern in a rat model of post-MI HF. Upregulation of miR-122-5p and miR-184 was found in HF rats, describing a proapoptotic role of both miRNAs (Liu et al., 2016). In another study using the same model, the authors identified a significant increase in miR-21-5p, miR-23a-3p, and miR-222-3p and their target *SOD2* in the plasma and myocardium of HF rats. They showed a direct interaction between miR-222-3p and *SOD2*. An inhibition or increase in *SOD2* expression was found when human cardiomyocytes were transfected with miR-222-3p mimic or inhibitor, respectively (Dubois-Deruy et al., 2017).

Myocardial fibrosis–related miRNAs, such as miR-19b, are reduced in the myocardium and serum of HF patients with aortic stenosis. Inhibition of miR-19b in cultured human fibroblasts increases the expression of connective tissue growth factor protein and the enzyme lysyl oxidase (LOX). This could lead to excessive collagen fibril cross-linking and a subsequent increase in LV stiffness in aortic stenosis patients, particularly those with HF. Thus, miR-19b could be a biomarker of alterations in the myocardial collagen network (Beaumont et al., 2017).

Numerous studies have been performed to find miRNAs with a predictive value in HF patients. Increased levels of miR-1, miR-21, miR-21-5p, miR-22-3p, miR-29a-3p, miR30d, miR-125a-5p, miR-125b-5p, miR-126-3p, miR-133b-3p, miR-195-3p, miR-197-5P, miR-208b-3p, miR-210-3p, miR-302b-3p, miR-320a, and miR-494-3p (Zhang et al., 2013b; He et al., 2017; van Boven et al., 2017; Wong et al., 2017; Xiao et al., 2017; Zhang et al., 2017a; Li et al., 2018a; Liu et al., 2018b;) or decreased levels of miR-17, miR-18a-5p, miR-20a, miR-150, miR-26b-5p, miR-27a-3p, miR-30e-5p, miR-106a-5p, miR-106b, miR-150-5p, miR-199a-3p, miR-423-5p, and miR-652-3p (Seronde et al., 2015; Ovchinnikova et al., 2016; Scrutinio et al., 2017; Shah et al., 2018a; Lin et al., 2019) have been described as potential biomarkers in HF patients. These discoveries may serve to develop miRNA-based therapies and to identify new pharmacological targets.

Beg et al. (2017) measured exosomal and total plasma miRNAs separately in HF patients to distinguish between the transfer of biological materials for signaling alteration in distant organs (exosomal) and the level of tissue damage (plasma). They found that the circulating exosomal miR-146a/miR-16 ratio was higher in HF patients, with miR-146a induced in response to inflammation. These results suggest circulating exosomal miR-146a as a biomarker of HF (Beg et al., 2017). Moreover, elevation of exosomal miRNA exo-miR-92b-5p has been suggested as a potential biomarker for the diagnosis of HF (Wu et al., 2018b; Wu et al., 2018c). In a preclinical study in dogs with myxomatous mitral valve disease, dysregulation of exosomal miR-9, miR-495, and miR-599 was observed as the dogs aged. In addition, levels of miR-9, miR-599, miR-181c, and miR-495 changed in myxomatous mitral valve disease. Thus, the exosomal miRNA expression level appears to be more specific to disease states than total plasma miRNA (Yang et al., 2017b). Furthermore, the downregulation of miR-425 and miR-744 in the plasma exosomes has been shown to induce cardiac fibrosis by suppressing TGFβ1 expression (Wang et al., 2018a).

Circulating miR-132 levels increased in chronic HF with disease severity, and lower levels improve risk prediction for HF readmission beyond traditional risk factors, but not for mortality. MiR-132 may be useful for finding strategies that would reduce rehospitalization in HF patients (Masson et al., 2018; Panico and Condorelli, 2018). Moreover, in an exhaustive analysis of two independent cohorts using a strict quality evaluation for miRNA testing, an association was found between high levels of miR-1254 and miR-1306-5p and mortality and HF hospitalization in HF patients. However, these two circulating miRNAs were not shown to improve standard predictors of prognostication, such as age, sex, hemoglobin, renal function, and NT-proBNP (Bayés-Genis et al., 2018).

MiR-26b, miR-208b, and miR-499 expression levels have been assessed in peripheral blood mononuclear cells from hypertensive HFpEF patients to evaluate their association with their exercise capacity. All three miRNAs were expressed at higher levels in the patients group, but miR-208b showed the strongest correlations with cardiopulmonary exercise test parameters, including oxygen uptake, exercise duration, and the minute ventilation–carbon dioxide production relationship (Marketou et al., 2018). In a study performed in patients and a mice model of hypertrophy and HF, miRNAs dysregulation was shown to occur during HF development in animals, with downregulation of target genes. These miRNAs were associated with adverse LV remodeling in humans, suggesting coordinated regulation of miRNA-mRNA. They also revealed target clusters of genes, such as autophagy, metabolism, and inflammation, implicated in HF mechanisms, (Shah et al., 2018b).

With the intention to establish a biomarkers panel useful for early detection of HF resulting from MI, Lakhani et al. (2018) found significant upregulation of miR-34a, miR-208b, miR-126, TGFβ-1, TNF-α, IL-6, and MMP-9 and reduced miR-24 and miR-29a levels. A positive association between IL-10 and ejection fraction in MI patients also suggested an important role of IL-10 in predicting HF (Lakhani et al., 2018).

Systems biology analyses of LV remodeling after MI allow molecular comprehensions; for example, miRNA modulation may be used as a marker of HF evolution. Two systems biology strategies were used to define an miRNA mark of LV remodeling in MI. They integrated either multiomics data (proteins and ncRNAs) produced from post-MI plasma or proteomic data generated from a rat model of MI. As a result, several miRNAs were associated with LV remodeling: miR-21-5p, miR-23a-3p, miR-222-3p, miR-17-5p, miR-21-5p, miR-26b-5p, miR-222-3p, miR-335-5p, and miR-375. These outcomes support the use of integrative systems biology analyses for the definition of miRNA marks of HF evolution (Charrier et al., 2019).

## Limitations and Perspectives of the Epigenetic Biomarkers

Limitations of the current field include the lack of large multicenter studies to provide convincing evidence for clinical applicability. Rather than a single ncRNA, it is likely that there will be patterns of different ncRNAs and other biomarkers (e.g., protein-based) that, together with machine-learning algorithms, will provide more sensitive and specific diagnostic and prognostic approaches to CVDs. Also, several technical challenges must be overcome before CE-marked ncRNA biomarkers will enter the clinical realm. DNA methylation and histone modifications are epigenetic mechanisms that have been reported to be sources of potential biomarkers useful in clinical practice. However, each CVD is regulated by multiple epigenetic pathways, and different CVDs are regulated by the same epigenetic mechanism, most of which are still under study. For example, hypermethylation of H3K79 (Rodriguez-Iturbe, 2006; Duarte et al., 2012) and *ACE2* promoter (Fan et al., 2017) in hypertensive patients has been described. Moreover, H3K4 and H3K9 were also hypermethylated in both mouse models of hypertension (Pojoga et al., 2011) and HF (Angrisano et al., 2014). This makes it difficult to select and implement a set of biomarkers for a particular CVD. Another potential problem is the quality of the samples, especially those obtained from collections in the pathology department. These samples are usually preserved in formaldehyde and paraffin, which highly degrades DNA. The stability, size, and integrity of a sample depend on the duration of fixation and storage (Kristensen et al., 2009). Thus, assessment of the quality of DNA is fundamental. However, the DNA methylation analysis can be performed successfully using polymerase chain reaction (PCR) methods with small amplicons in old samples (Tournier et al., 2012; Wong et al., 2014). In other cases, it is important to carefully adjust the protocol. It is also important to consider that frozen and paraffin-preserved samples may have different results, and they should not be compared without appropriate correction (García-Giménez et al., 2017).

Among the epigenetic biomarkers, miRNAs are the most promising, and numerous studies have been carried out in the last few years. The relatively easy detection and accessibility to samples in fluids, such as blood, urine, or saliva, make them very attractive. However, a few issues should be solved before their implementation in the clinical practice. The main problem is that miRNAs usually target multiple mRNAs from different genes, and one gene can be targeted by several miRNAs. This complex network should be deeply investigated before determining the use of a specific miRNA as a biomarker for the diagnosis or treatment of a particular disease (Akhtar et al., 2016). Regarding sample preparation, it is highly recommended to use plasma instead of whole blood, because if it is hemolyzed, the circulating miRNA content can be altered. Increasing the centrifugation time is also important in order to reduce platelet contamination (de Gonzalo-Calvo et al., 2017; García-Giménez et al., 2017).

Recently, great advances have been made to implement the new technology in the detection of new epigenetic biomarkers. However, a few concerns should be alleviated before their clinical implementation. Studies with big cohorts in different independent laboratories, using the same experimental design, sample preparation, methodology, and disease specifications, are necessary. Small patient cohorts should be considered as pilot studies before the validation of results in bigger sample analysis. The method of detection should be standardized for clinical application, and the clinical trials have to be randomized and prospective. It is also important to compare the new biomarkers with the classical biomarkers in order to validate them and determine their usefulness. The sensitivity and specificity for a certain disease also have to be determined for each biomarker (Engelhardt, 2012; García-Giménez et al., 2017). Regarding the method of DNA methylation detection, the luminometric methylation assay and the methylation analysis of CpG islands in repeatable elements (LINE-1) are widely used. Although there is a certain correlation with the measurements obtained with both methods, the comparison is not recommended, since a consistent bias between the results has been described (Knothe et al., 2016). Interestingly, a large multicenter study comparing DNA methylation assays compatible with routine clinical use has been performed. According to the authors, good agreement was observed between DNA methylation assays, which can be implemented in large-scale validation studies, development of new biomarkers, and clinical diagnostics (BLUEPRINT Consortium, 2016). The most used system to detect miRNAs is quantitative PCR, being the normalization protocol critical. Most laboratories use housekeeping genes or miRNAs as normalizers, changing their expression levels within serums. Another approach employs identical volumes of serum for all samples, generating different amounts of total RNA (Chen et al., 2008; Wang et al., 2009; Rockenbach et al., 2012). Both approaches include spike-in normalization, which consists of adding RNA of known sequence and quantity to calibrate measurements. However, spike-in normalization does not consider internal variation in circulating miRNA between different individuals. Thus, a combination of both methods should always be performed to guarantee results reliability (van Empel et al., 2012). Polymerase chain reaction technology has to be performed with rigorous controls to avoid artifacts in the amplification step. To overcome this problem, digital PCR based on the amplification of one single molecule per reaction constitutes a valuable option (Hindson et al., 2013). Another attractive alternative for accurate measuring RNAs is the direct nucleic acid sequencing, although it is still expensive when considering large screening analysis (Kozomara and Griffiths-Jones, 2011). Finally, it is also important to understand the processes controlling miRNAs release and stability. The correlation between circulating and tissue miRNAs is not clear, and several studies indicate that miRNA levels in blood are not a reflection of changes in the tissue of origin. The reason is that miRNAs can also be produced by immune cells (Zheng et al., 2018).

## Concluding Remarks

Over the past few years, a great amount of research has focused on epigenetics and its dynamic cross-talk with genetics. Unveiling a personalized epigenetic pattern can provide a large amount of information on epigenetic machinery that could be employed to tailor diagnosis and therapeutic strategies in CVDs. Recent advances in technology and data analysis have made it possible to create detailed epigenetic maps, which may represent a new tool in the clinical practice to discern cardiovascular risk beyond traditional risk determinants. Epigenetic information can also help in predicting individual drug responses. Importantly, epigenetic biomarkers are gaining ground in the scientific community as tools for the diagnosis and prognosis of CVDs. However, discrepancies in specific diagnostic biomarkers make replication of the current results in independent laboratories, with multiple research centers and a big sample size, mandatory. All of this will lead to a standardized clinical application in the near future.

## Author Contributions

CS-B and AB-G conceived the idea and wrote the manuscript with support from CG-M. CG-M performed the drawings and structure of the figures. All authors contributed to manuscript revision, read and approved the submitted version.

## Funding

This work was supported by grants from the Spanish Ministry of Economy and Competitiveness-MINECO (SAF2017-84324-C2-1-R), the Instituto de Salud Carlos III (PIC18/0014, PI18/00256), the Red de Terapia Celular–TerCel (RD16/0011/0006) and the CIBER Cardiovascular (CB16/11/00403) projects, as part of the Plan Nacional de I+D+I, and it was co-funded by ISCIII-Sudirección General de Evaluación y el Fondo Europeo de Desarrollo Regional (FEDER). This work was also funded by the Fundació La MARATÓ de TV3 (201516-10, 201502-20), the Generalitat de Catalunya (SGR2017 00483, SLT002/16/00234), the CERCA Programme/Generalitat de Catalunya, and “la Caixa” Banking Foundation.

## Conflict of Interest

The authors declare that the research was conducted in the absence of any commercial or financial relationships that could be construed as a potential conflict of interest.

## Abbreviations

AMI, acute myocardial infarction; ApoE, apolipoprotein E; BNP B-type natriuretic peptide; CK, creatine kinase; cTnI, cardiac troponin I; cTnT, cardiac troponin T; DOT1L, disruptor of telomeric silencing-1; ENaC, epithelial sodium channel; EZH2, enhancer of zeste homolog 2; GEO, Gene Expression Omnibus; HDAC, histone deacetylase; HF, heart failure; HFrEF, heart failure with reduced ejection fraction; HFpEF, heart failure with preserved ejection fraction; hs-cTnT, high-sensitivity cardiac troponin T; hs-CRP, high-sensitivity C-reactive protein; lncRNAs, long noncoding RNAs; LV, left ventricular; MI, myocardial infarction; miRNAs, microRNAs; ncRNAs, noncoding RNAs; NSTEMI, non- ST-segment elevation myocardial infarction; STEMI, ST-segment elevation myocardial infarction; pmiRNAs, platelet miRNAs; piRNAs, p-element-induced wimpy testis (PIWI)-interacting RNAs; tRNA, transfer RNA; ZEB1, zinc finger E-box binding homeobox 1.
